# Tribological investigations of the load, temperature, and time dependence of wear in sliding contact

**DOI:** 10.1371/journal.pone.0175198

**Published:** 2017-04-20

**Authors:** Matthew David Marko, Jonathan P. Kyle, Yuanyuan Sabrina Wang, Elon J. Terrell

**Affiliations:** 1 Department of Mechanical Engineering, Columbia University, 500 W 120 St, New York, NY 10027 United States of America; 2 Naval Air Warfare Center Aircraft Division, Joint-Base McGuire-Dix-Lakehurst, Lakehurst NJ 08733 United States of America; 3 Blue Origin, Kent WA United States of America; 4 Foxconn, Tucheng District, New Taipei, Republic of China (Taiwan); 5 Sentient Science Corporation, 672 Delaware Avenue, Buffalo NY 14209 United States of America; Beihang University, CHINA

## Abstract

An effort was made to study and characterize the evolution of transient tribological wear in the presence of sliding contact. Sliding contact is often characterized experimentally via the standard ASTM D4172 four-ball test, and these tests were conducted for varying times ranging from 10 seconds to 1 hour, as well as at varying temperatures and loads. A numerical model was developed to simulate the evolution of wear in the elastohydrodynamic regime. This model uses the results of a Monte Carlo study to develop novel empirical equations for wear rate as a function of asperity height and lubricant thickness; these equations closely represented the experimental data and successfully modeled the sliding contact.

## Introduction

Friction and wear is a problem that affects practically every field of engineering. Wear has the effect of reducing the life of materials, and causing eventual failures of the mechanical systems. In practically any mechanical system, any friction ends up as lost energy, reducing the overall efficiency. Finally, friction can cause runaway heating that can damage or destroy mechanical components.

The tribological phenomena of wear and friction is an essential design consideration for practically every mechanical device. Most of this friction is transient, occurring over an extended period of time throughout the life of the mechanical device. Friction is a dynamic and nonlinear process as the shape at the point of contact changes from the material wear; therefore it is necessary to understand the transient wear rates and the phenomena of running-in, the tribological process of friction dynamically reaching steady-state as the wear evolves in time.

Running-in is a tribological phenomenon characteristic of the physical, chemical, and geometric characteristics of the contact surface [1–14]. With this in mind, it can be clearly stated that wear rate $\dot{V}$ (m^3^/s) is a function of the existing wear *V* (m^3^). While there are several phenomena that can cause wear, one of the most profound causes are asperities in the surface. One established equation to represent wear resulting from adhesion and abrasion is the Archard’s equation [3, 15]

$$\begin{array}{ccc} V & = & K_{wear}\cdot \frac{W\cdot S}{H}, \end{array}$$ (1)

where *W* (Newtons) is the contact load, *S* (m) is the sliding distance, *H* (Pa) is the material hardness, and *K*_*wear*_ (dimensionless) is the wear coefficient for a steady wear rate.

This current form of Archard’s equation in Eq 1 is only representative of the wear trend; a wear model requires either extensive Monte Carlo simulations [16] or a substantial amount of prior wear data [17] to fit into these equations. One example of the limitation of this equation is that there is no clear consensus on the relationship between wear rate and both the load and the hardness; while increasing load and / or decreasing the material hardness will inherently increase the wear, the relationship is not necessarily linear [1, 18, 19]. As described in reference [2], the only tribological parameters that will have a linear relationship on the wear is the area of contact, the speed of sliding contact, and the average height of the surface asperities

$$\begin{array}{ccc} \dot{V} & = & V_{n}\cdot \sigma \cdot U\cdot \Delta x, \end{array}$$ (2)

where *σ* (m) is the RMS surface roughness, Δ*x* (m) is the width of the region of contact perpendicular to the velocity *U* (m/s), and *V*_*n*_ is a dimensionless wear rate, proportional to several dimensionless ratios

$$\begin{array}{ccc} V_{n} & \propto & f(\frac{\sigma }{h},\frac{P}{H},\kappa_{ellipse}), \end{array}$$ (3)

where *h* (m) is the lubricating oil thickness, *P* (Pa) is the pressure from the load, and *κ*_*ellipse*_ is the ellipticity of the area of contact [18–20].

It is desired to develop a practical numerical method of modeling and simulating the phenomena of abrasive wear, caused by asperities in sliding contact, without needing substantial empirical data to start with. Such a method can be used to reduce the need for repetitive four-ball tests, which require expensive equipment and are time-consuming to perform. A reliable numerical model will help to better understand analytically and conceptually the phenomena of wear evolution, to improve on practical engineering design.

## Film thickness model

**Fig 1:**
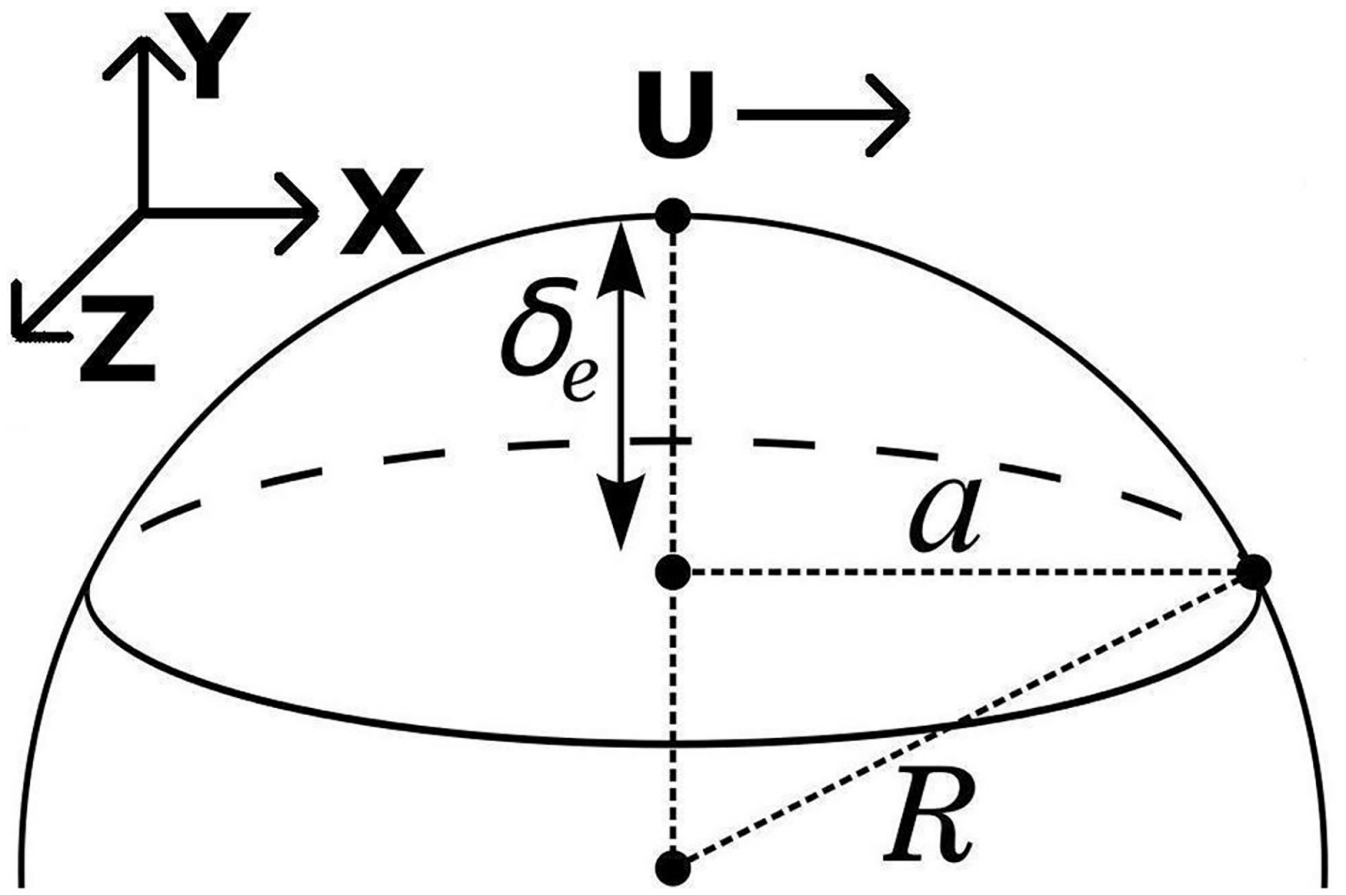
Definitions for indentation function defined by Eq 4, and the definition of the *x*, *y*, and *z* dimensions.

A numerical model was developed [2] to solve the Archard’s equation and determine the wear rate as it is distributed over the area in contact. To do this, it is clear that the wear rate is strongly proportional to the film thickness, and therefore it is necessary to realize it [18, 21–27] in order to properly predict the wear rate. The first step is to break down the area of contact into a defined two-dimensional (2D) meshed grid. The equation for the indentation of the ball bearing is [2]

$$\begin{array}{ccc} F_{indent}\left(x,z,R\right) & = & 2\cdot R\cdot \left[1-cos\left(sin^{-1}\frac{\sqrt{x^{2}+z^{2}}}{R}\right)\right], \end{array}$$ (4)

where *R* (m) is the radius of the ball bearing. Eq 4 can be derived by the trigonometric relationships described in Fig 1. It is safe to assume that throughout the entire domain of the ball bearing, surrounding the area of contact, the surface is entirely immersed in oil. With this assumption, the lubricant oil film thickness will comprise of the sum total of the profile of the ball bearing *F*_*indent*_ (m), elastic deflection from the pressure of contact *δ*_*e*_ (m), any wear that may have previously occurred *V*_*y*_ (m) [28], as well as the minimum elastohydrodynamic lubricant thickness *h*_*min*_ (m).

**Fig 2:**
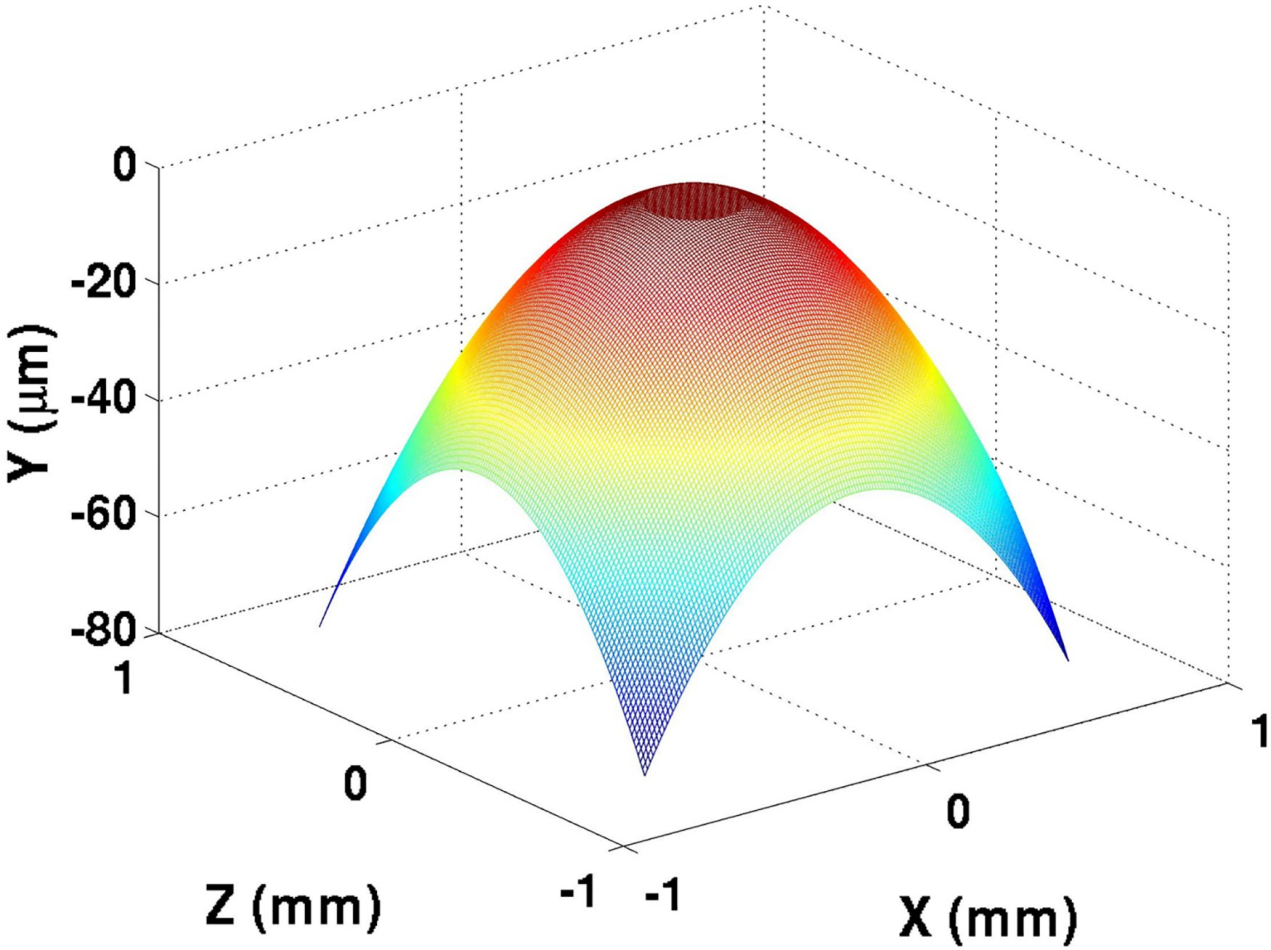
Ball bearing profile subjected to Hertzian deflection for 391 Newtons of load. The Hertzian pressure function (Eq 7) was divided by the Winkler Mattress coefficient (Eq 6), and the deflection yielded a flat surface at the region of contact.

The next step to estimating the lubricant film thickness is to calculate the elastic deformation as a result of the lubricant pressures. To determine this deflection, the Winkler Mattress model is assumed [28], where the deflection at each finite difference node is linearly proportional to the pressure following Hooke’s Law; the deflections are small compared to the total length and thus there are no significant shearing forces. The elastic deflection *δ*_*e*_ (m) can be easily calculated as

$$\begin{array}{c} \delta_{e}=\frac{P}{K_{h}}, \end{array}$$ (5)

where *P* (Pa) is the pressure; and *K*_*h*_ (Pa/m) is the Winkler Mattress coefficient. While there are several approaches to calculating *K*_*h*_ [28], this model calculates it by comparing the estimated Hertzian pressure to the estimated Hertzian deflection, where

$$\begin{array}{c} K_{h}=\frac{P_{Hertz}}{\delta_{Hertz}}, \end{array}$$ (6)

where *P*_*Hertz*_ (Pa) is the maximum Hertzian pressure, and *δ*_*Hertz*_ (m) is the maximum Hertzian deflection [9, 20], both for dry, no-wear, elastic contact, and with these terms the Winkler Mattress coefficient can be determined from Eq 6

$$\begin{array}{c} P_{Hertz}=\frac{3}{2}\frac{W_{0}}{\pi a_{Hertz}^{2}},\delta_{Hertz}={\left(\frac{9}{16}\frac{W^{2}}{R^{\prime }\cdot E^{\prime 2}}\right)}^{\frac{1}{3}},a_{Hertz}={\left(\frac{3WR^{\prime }}{2E^{\prime }}\right)}^{\frac{1}{3}},\\ R^{\prime }=\frac{R}{2},E^{\prime }=\frac{E}{1-p^{2}},r=\sqrt{x^{2}+z^{2}}, \end{array}$$ (7)

and by imposing this pressure with the Winkler Mattress coefficient determined in Eq 6, a flat profile can be observed within the radius contact in Fig 2.

Due to the presence, however, of both the lubricant oil as well as the previous wear on the ball bearing profile, Eq 7 cannot be assumed for the pressure. The Reynolds equation must be solved [28] in order to get the true lubricant oil pressure and deflection. Within the Reynolds equation, the film thickness will directly affect the pressure function, which affects the elastic deformation, which affects the pressure. For this reason, an iterative solver [2, 29–33] will be needed to converge on a solution of both the pressure and the film thickness in the presence of the ball bearing profile, previous wear, and the minimum elastohydrodynamic film thickness.

In addition to the pressure and elasticity, the minimum elastohydrodynamic lubrication film thickness needs to be realized. This is a small amount of oil, typically 1 *μ*m thick [20] or less, subjected to extreme pressures from the contact. One cause of this minimum lubricant thickness is from hydrodynamic film formation, such as boundary layer and other effects from simple hydrodynamic lubrication. A second cause of this minimum thickness is modification of the material geometry; the two surfaces deform elastically to form a quasi-parallel region for the lubricant to flow through. Finally, according to Barus law [20, 34], the viscosity increases exponentially with pressure

$$\begin{array}{ccc} \nu_{P} & = & \nu_{0}\cdot e^{\alpha_{P}P}, \end{array}$$ (8)

where *P* (Pa) is the pressure, *ν*_*P*_ and *ν*_0_ (m^2^/s) are the kinematic viscosity under high and atmospheric pressure respectively, and *α*_*P*_ (Pa^−1^) is the pressure-viscosity coefficient (PVC) of the lubricant, [20, 35]. Under the extreme pressures that occur at the point of contact, the viscosity can increase dramatically, and also contribute to the overall lubricant film thickness; this is the very definition of elasto-hydrodynamic contact.

There are numerous prior studies for the lubricant oil film thickness [2, 19, 21–27], though one of the most versatile is the study conducted by Hamrock and Dowson [18]. Film thickness profiles were studied experimentally for a large series of elastohydrodynamic profiles for varying dimensions, and optical interferometry was used to measure both the minimum and central film thickness. They used a variety of different materials, lubricants, speeds, loads, and contact dimensions, to come with a single empirical solution for the lubricant oil thickness. The Hamrock-Dowson equations for the minimum and central film thickness [18]

$$\begin{array}{c} h_{min}=3.63\cdot R^{\prime }\cdot \left(U_{n}^{0.68}\right)\cdot \left(G_{n}^{0.49}\right)\cdot \left(W_{n}^{-0.073}\right)\cdot \left(1-exp[-0.68\kappa_{ellipse}]\right),\; \end{array}$$ (9)

$$\begin{array}{c} h_{c}=2.69\cdot R^{\prime }\cdot \left(U_{n}^{0.67}\right)\cdot \left(G_{n}^{0.53}\right)\cdot \left(W_{n}^{-0.067}\right)\cdot \left(1-0.61\cdot exp[-0.73\kappa_{ellipse}]\right),\; \end{array}$$ (10)

$$\begin{array}{c} U_{n}=\frac{\mu_{0}\cdot U}{E^{\prime }\cdot R^{\prime }},\; \end{array}$$ (11)

$$\begin{array}{c} G_{n}=\alpha_{PVC}\cdot E^{\prime },\; \end{array}$$ (12)

$$\begin{array}{c} W_{n}=\frac{W}{E^{\prime }\cdot R^{\prime 2}},\; \end{array}$$ (13)

where *h*_*min*_ (m) is the minimum film thickness, *h*_*c*_ (m) is the central film thickness, *U*_*n*_ is the dimensionless speed parameter, *G*_*n*_ is the dimensionless material parameter, *W*_*n*_ is the dimensionless load parameter, *κ*_*ellipse*_ is the ellipticity of the contact area, *μ*_0_ (Pa-s) is the dynamic viscosity of the lubricant at atmospheric pressure, and *U* (m/s) is the velocity of sliding contact of the four-ball test. It is clear that before the pressure and film thickness profile can be realized, it is necessary to determine the dynamic viscosity and the minimum film thickness, so that a proper film thickness function can be realized and the wear rate analyzed.

## Viscosity calculations

In order to realize the minimum elastohydrodynamic film thickness, it is necessary to determine the dynamic viscosity of the lubricant. The viscosity of the lubricant, however, is affected by temperature [2, 28, 35–38], as hotter oils are inherently less viscous. A reduction in viscosity results in a reduced minimum film thickness [18], but this reduced film thickness results in a cooler oil film [39], as there is less thermal resistance from the center of the oil film to the surface of the ball bearing. As a result of this contradiction, it is necessary to use iteration in order to converge on a realistic lubricant oil temperature and viscosity, so that a minimum film thickness can be determined.

As described in reference [2], the first step is to calculate the flash temperature heating of the surface of the ball bearing. This is done by first calculating the dimensionless Peclet number [20, 39]

$$\begin{array}{ccc} L & = & \frac{U\cdot a}{2\alpha_{bb}}, \end{array}$$ (14)

where *a* is the radius of the area of contact, and *α*_*bb*_ (m^2^/s) is the thermal diffusivity [40] of the ball bearing

$$\begin{array}{ccc} \alpha_{bb} & = & \frac{k_{bb}}{\rho_{bb}\cdot C_{P,bb}}, \end{array}$$ (15)

where *k*_*bb*_ (*W*/*m*^2^⋅°C) is the thermal conductivity, *ρ*_bb_ (kg/m^3^) is the density, and *C*_*P*,*bb*_ (J/kg⋅°C) is the specific heat capacity; all of these parameters are for the ball bearing material (steel).

The predictive analytical equation used by this model for average flash temperature can vary with Peclet number, where [20, 39]

$$\begin{array}{ccc} \Delta T_{F}= & \frac{\mu_{COF}\cdot W\cdot U}{4\cdot k_{bb}\cdot a} & L<0.1,\\ \Delta T_{F}= & \left[0.35+\left(5.0-L\right)\frac{0.5}{4.9}\right]\frac{\mu_{COF}\cdot W\cdot U}{4\cdot k_{bb}\cdot a} & 0.1<L<5.0,\\ \Delta T_{F}= & \frac{0.308\mu_{COF}\cdot W\cdot U}{4\cdot k_{bb}\cdot a}\sqrt{\frac{\alpha_{bb}}{U\cdot a}} & L>5.0, \end{array}$$ (16)

where *μ*_*COF*_ is the dimensionless coefficient of friction (COF), *W* (Newtons) is the load, and Δ*T*_*F*_ (°C) is the surface temperature increase due to friction. In the case of the steel ball bearings, the friction coefficient is *μ*_*COF*_ = 0.10 (experimentally realized), the thermal conductivity *k*_*bb*_ = 46.6 W/m^2^⋅°C, the density *ρ*_*bb*_ = 7,810 kg/m^3^, the specific heat capacity *C*_*P*,*bb*_ = 475 J/kg⋅°C, and the thermal diffusivity *α*_*bb*_ = 12.56 mm^2^/s.

The next step in realizing the elastohydrodynamic film thickness is to estimate the temperature increase of the lubricant as a result of the friction heating. This field was investigated extensively for helical gears [41] and square contact surfaces seen in cutting tools [42], and these classic theories were adjusted for circular contact by Archard in 1958 [39]. Archard’s work focused on time-dependent flash heating to match experimental studies conducted by Crook [43], and an equation for the lubricant oil temperature increase Δ*T*_*L*,0_ (°C) at the center of the film ($y=\frac{h}{2}$) [39]

$$\begin{array}{c} \Delta T_{L,0}=\left(\frac{q_{v}h^{2}}{8k_{lub}}\right)\left[1-\frac{32}{\pi^{3}}\Sigma_{m=0}^{m=\infty }\frac{{\left(-1\right)}^{m}}{{\left(2m+1\right)}^{3}}\left(exp-\frac{\alpha_{lub}{\left(2m+1\right)}^{2}\pi^{2}t}{h^{2}}\right)\right], \end{array}$$ (17)

where *q*_*v*_ (Watts/m^3^) is the friction energy generated per unit volume, *h* (m) is the film thickness, *k*_*lub*_ (Watts/m-°C) is the thermal conductivity of the lubricant, and *α*_*lub*_ (m^2^/s) is the thermal diffusivity [40] of the oil

$$\begin{array}{ccc} \alpha_{lub} & = & \frac{k_{lub}}{\rho_{lub}\cdot C_{P,lub}}, \end{array}$$ (18)

where *ρ*_*lub*_ (kg/m^3^) is the density of the lubricant, and *C*_*P*,*lub*_ (J/kg⋅°C) is the specific heat capacity of the lubricant.

The lubricant model being developed will assume steady-state heating, as the time-steps are longer than the flash temperature durations. This can be verified by determining when the first exponential term in the series in Eq 17 reaches 1%. Assuming a film thickness of *h* = 1 *μ*m and a thermal diffusivity of *α*_*lub*_ = 7.73⋅10^−8^ m^2^/s, the flash temperature increase reaches steady state

$$\begin{array}{ccc} t_{ss} & = & -\frac{log_{N}\left(0.01\right)h^{2}}{\alpha_{lub}\pi^{2}}, \end{array}$$ (19)

at *t*_*ss*_ = 6 *μ*s. This is far shorter than any time-step in the simulations, and therefore the model will treat the lubricant oil temperature increase as the result of steady-state conductive heat transfer from the center of the lubricant film to the surface of the ball bearing.

The steady-state conductive heat transfer equation [40] with heat generation from friction heating is

$$\begin{array}{ccc} \frac{d^{2}T_{L}}{dy^{2}} & = & \frac{q_{v}}{k_{lub}}, \end{array}$$ (20)

and thus the temperature profile of the lubricant *T*_*L*_(*y*) (°C) is

$$\begin{array}{ccc} T_{L}\left(y\right) & = & \frac{q_{v}}{2\cdot k_{lub}}\left[\left(h\cdot y\right)-y^{2}\right]+T_{surface}, \end{array}$$ (21)

where *y* (m) is the film thickness position, and *T*_*surface*_ (°C) is the surface temperature

$$\begin{array}{ccc} T_{surface} & = & \Delta T_{F}+T_{B}, \end{array}$$ (22)

where Δ*T*_*F*_ (°C) is the surface temperature increase in Eq 16, and *T*_*B*_ (°C) is the bulk lubricant temperature. It is clear that Eq 21 is simply the steady-state (*t* = ∞) solution Eq 17. Averaging Eq 21 over the depth of the film thickness (0 < *y* < *h*), an average lubricant temperature *T*_*L*_ (°C) can be found as

$$\begin{array}{ccc} T_{L} & = & 0.1665\frac{q_{v}}{k_{lub}}\frac{h^{2}}{2}+T_{surface}. \end{array}$$ (23)

The next step is to determine the volume rate of heat energy *q*_*v*_ (Watts/m^3^) being dissipated into the oil from the friction heating. The friction heat energy density is assumed to be the total of the friction forces being dissipated into the lubricant, as a function of the volume of oil covering the area of contact. The power into the oil *Q*_*lub*_ (Watts) is a function of the product of the friction forces and the velocity

$$\begin{array}{ccc} Q_{lub} & = & \mu_{COF}\cdot W\cdot U, \end{array}$$ (24)

where *μ*_*COF*_ is the dimensionless COF, *W* (Newtons) is the load, and *U* (m/s) is the velocity of sliding contact. The volume of the oil *V*_*lub*_ (m^3^) is simply the product of the area of contact and the film thickness *h* (m)

$$\begin{array}{ccc} V_{lub} & = & h\cdot \pi a^{2}, \end{array}$$ (25)

where *a* (m) is the radius of contact. With these two values, the rate of heating per volume *q*_*v*_ (Watts/m^3^) can be determined

$$\begin{array}{ccc} q_{v} & = & \frac{\mu_{COF}\cdot W\cdot U}{h\cdot \pi a^{2}}, \end{array}$$ (26)

and with a value of *q*_*v*_, the final average lubricant temperature *T*_*L*_ (°C) of the oil film

$$\begin{array}{ccc} T_{L} & = & 0.1665\frac{\mu_{COF}\cdot W\cdot U}{\pi a^{2}}\frac{h}{2k_{lub}}+T_{surface}. \end{array}$$ (27)

This lubricant temperature *T*_*L*_, heated by the friction of sliding contact, can be used to determine the lubricant viscosity [20, 38, 44], which is a necessary parameter to determine the film thickness with the Hamrock-Dowson [18] empirical equations.

According to Eq 27, it is clear that the oil temperature increase is linearly proportional to the film thickness; while Eq 9 shows how a decrease in viscosity (such as from an increase in temperature) would reduce the film thickness. For this reason, iteration is needed to converge on a final lubricant temperature, viscosity, and minimum film thickness. The Hamrock-Dowson [18] empirical equation for the central film thickness (Eq 10) can be used as an approximate central film thickness to attempt to iterate for a new temperature and viscosity. This iterative loops repeats itself until it converges at a final value for the lubricant oil temperature and viscosity. The final viscosity can be used in Eq 9 for a minimum film thickness value in order to find the full film-thickness function.

## Numerical solution of the Reynolds equation

The Reynolds equations is a well established differential equation derived from the Navier-Stokes equation to predict the pressure distribution in a lubricating film separating two surfaces in contact [20, 28]. The general form of the Reynolds equation is

$$\begin{array}{c} \frac{\partial }{\partial x}\left(\frac{\rho h^{3}}{\mu }\cdot \frac{\partial P}{\partial x}\right)+\frac{\partial }{\partial z}\left(\frac{\rho h^{3}}{\mu }\cdot \frac{\partial P}{\partial z}\right)=\frac{\partial }{\partial x}\left[6\rho hU_{x}\right]+\frac{\partial }{\partial z}\left[6\rho hU_{z}\right]+12\frac{d}{dt}\left(\rho h\right),\; \end{array}$$ (28)

where *U*_*x*_ and *U*_*z*_ (m/s) are the flow velocities in and out of the thin-film boundary in the *x* and *z* direction (see Fig 1), *P* (Pa) is the pressure, *h* (m) is the film thickness, and *μ* (Pa-s) is the dynamic viscosity.

**Fig 3:**
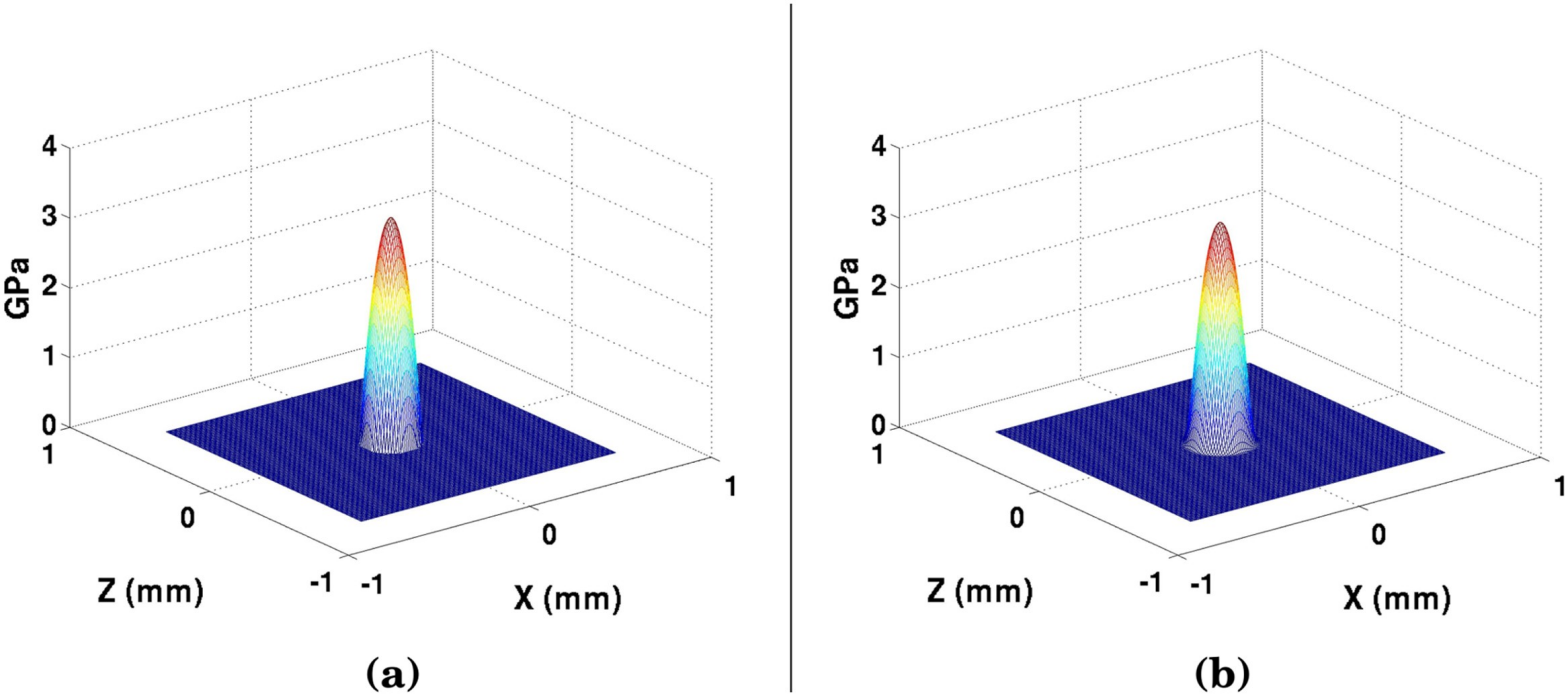
(a) Hertzian pressure distribution from Eq 7, and (b) lubricant oil pressure with no-wear.

The next step is to discretized the Reynolds equations, including the pressure distribution (ex. Fig 3). By using using Taylor-Series expansion to discretize the pressure [45], the Reynold’s equation can be described as a 2D series of finite difference nodes. One challenge that must be overcome in this effort is the fact that Barus Law breaks down for the high-pressures greater than 500 MPa [28], and therefore the Grubin model [20, 46–49] will not be applicable. Since the region of contact can see pressures on the order of GPa, the viscosity-pressure relationship is found with the *Roelands* equation [28, 50]. The discrete Reynold’s equation can then be used to find the pressure distribution as a function of the lubricant film thickness.

**Fig 4:**
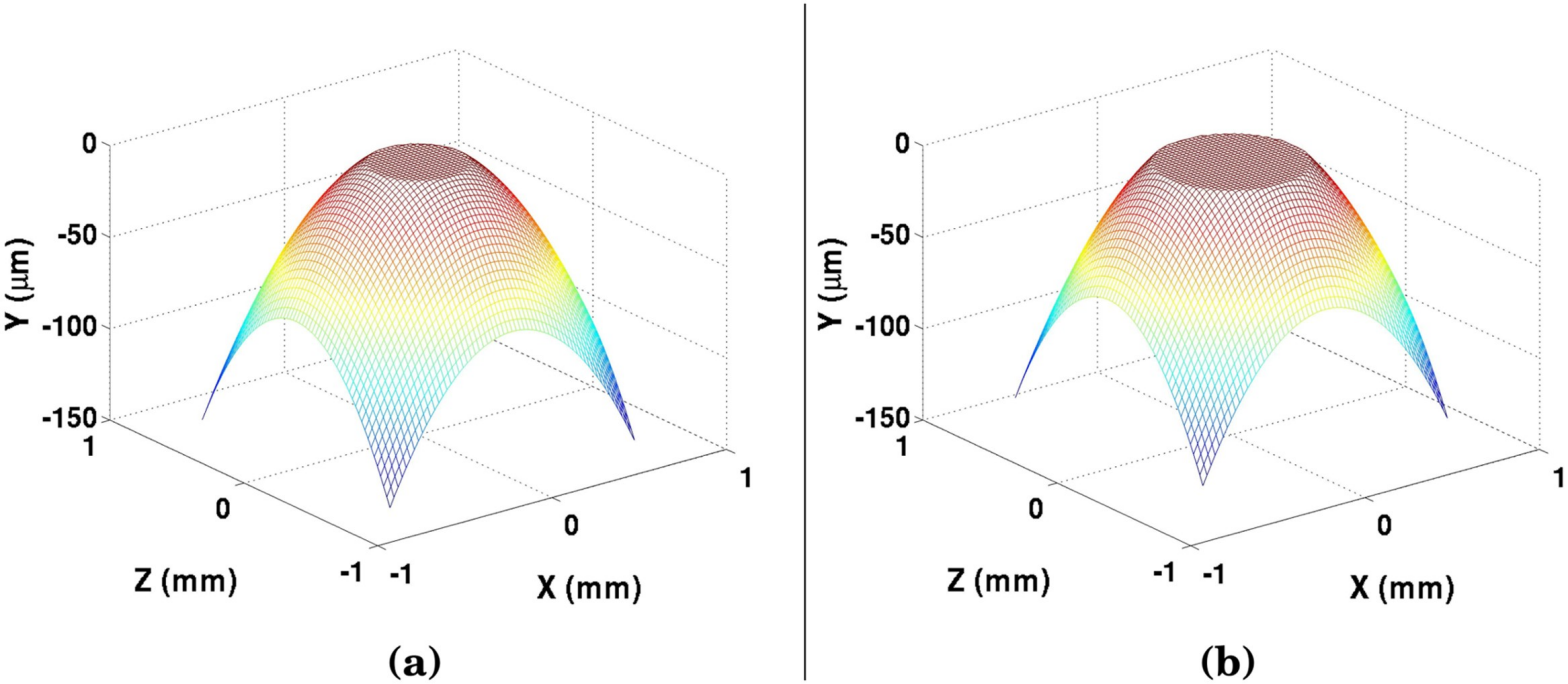
Film thickness profile after 3600 seconds of contact, at both (a) 25°C and (b) 59°C.

The convergence of the pressure distribution for a given film thickness is not necessarily a final solution for the pressure. A change in pressure would yield a change in elastic deformation, which would further alter the pressure profile. After the first pressure convergence, the new pressure is used to find a new profile of the elastic deformation based on the Winkler Mattress Eq 5, and a new film thickness profile is developed. The film profile is normalized to the minimum film thickness realized in Eq 9, and the pressure iteration is repeated. This process repeats itself until the pressure, elastic deformation, and lubricant oil film thickness converge for the given ball-bearing profile and prior wear. Overwhelmingly with wear, the film thickness profile will appear flat (Fig 4). Once the proper film thickness profile is determined, the wear rate can be predicted for the next time-step.

## Wear simulations

The most important part of this simulation is to figure out the sliding contact wear rate. The first value to realize is the velocity, which is a specified parameter of the four-ball test; the hardness, which is an experimentally realized material parameter; and the pressure, which is determined with iteration and the Reynolds equation. These terms are only proportional, and a relationship between these values and the true wear rate must be realized.

As observed in Eq 2 [15], this wear is related to the ratio of the surface roughness over the lubricant thickness. The principle action of wear in the elastohydrodynamic regime [2, 20, 28] occurs when the material asperities exceed the thickness of the lubricant [1, 2, 15, 51–55]; hence the larger and thicker the asperity, the greater the wear. Certainly it is not possible to model every single asperity with infinite accuracy, but a root mean squared (RMS) value of the fluctuation of the surface can be easily measured and characterized optically. For the highly polished, test-grade ball bearings used in four-ball tests, where the surface roughness is less than optical wavelengths, this assumption of a normal distribution is necessary. By definition, the RMS value of the asperities assumes a normal distribution for the probability of a given peak reaching a certain height.

One important consideration to calculating the wear rate is the material hardness, especially the yield stress in shear, as wear occurs when the shear stresses exceed the ultimate yield stress and material is lost. It is intuitively obvious that not all asperities that come into contact with the sliding surface will necessarily be lost as wear; some asperities will only experience elastic deflection. To get around this, a plasticity or yield length needs to be determined, where [1, 2]

$$\begin{array}{ccc} W_{P} & = & R^{\prime }\cdot {\left(\frac{G_{yield}}{E^{\prime }}\right)}^{2}, \end{array}$$ (29)

where *R’* (m) is the reduced radius (Eq 7) of the ball bearing, *G*_*yield*_ (Pa) is the ultimate yield stress, *E’* (Pa) is the reduced Young’s modulus, and *W*_*P*_ (m) is the yield / plasticity length.

Wear occurs when a random asperity exceeds both the film thickness height plus the yield length from Eq 29. This can be characterized as the dimensionless λ_*W*_-value [2]

$$\begin{array}{ccc} \lambda_{W} & = & \frac{h+W_{P}}{\sigma }, \end{array}$$ (30)

and this parameter is proportional to the wear according to Archard’s Wear equation [15]. Wear would occur whenever a random asperity exceeds a certain λ_*W*_-value, which represents the ratio of roughness standard deviations that contact occurs. The lower the λ_*W*_-value, the higher the probability of an asperity exceeding this film thickness height, and thus the more wear would occur.

**Fig 5:**
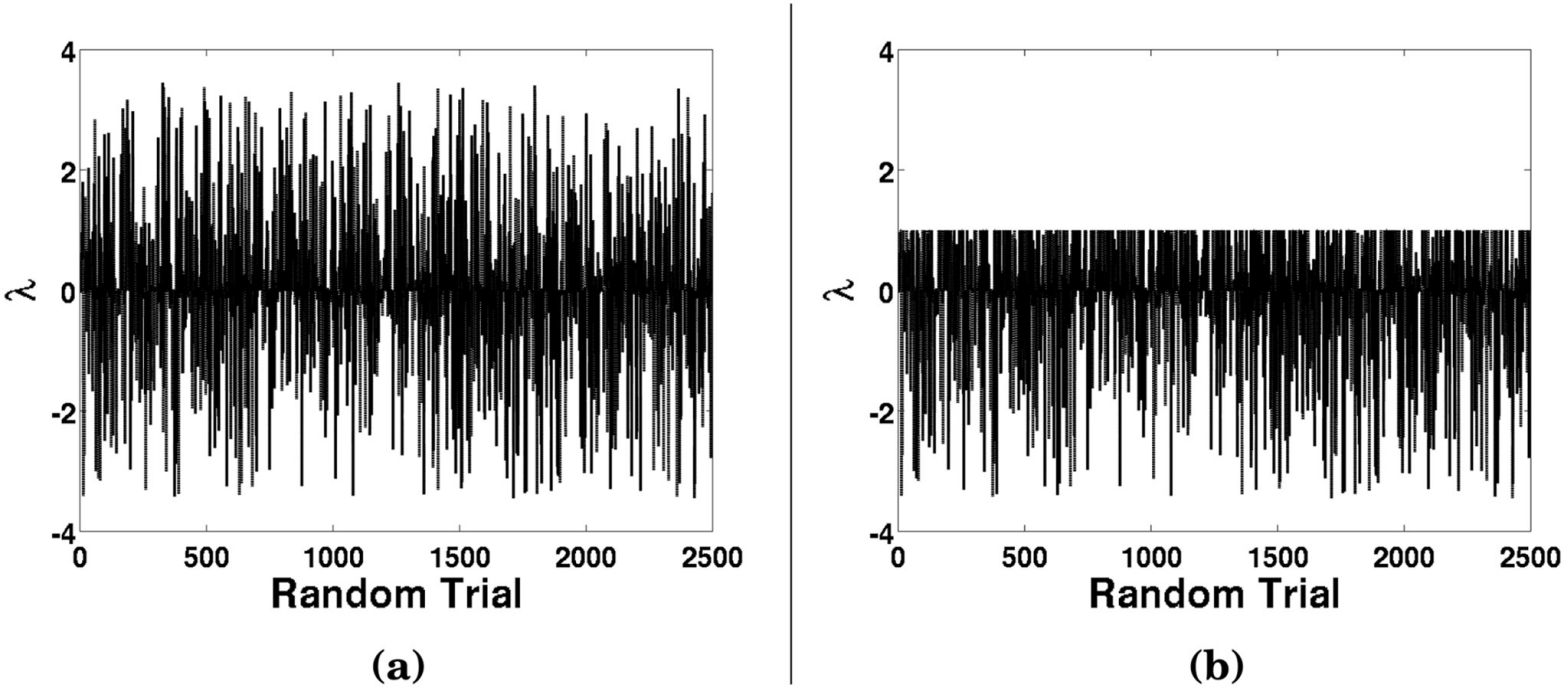
Monte Carlo data of random normalized asperities, both (a) before any wear and (b) after λ_*W*_ = 1 of contact.

A Monte Carlo simulation was conducted to attempt to predict the expected wear that would occur from a given λ_*W*_-value, which will remove all the asperities that exceed a given ratio of standard deviations. The reason for this approach, as opposed to assuming the asperities height follows a normal or Gaussian distribution, is to be able to develop an exponential decaying function, which is expected according to reference [1], when only an RMS asperities height can be realistically measured, as is the practical case when measuring the surface roughness of test grade ball bearings with optical profilometry. The asperities were represented by *N* = 10^9^ random numbers ranging from -1 to 1 (Fig 5-a), and the standard deviation of this sequence was determined. The random sequence generated with MATLAB was raised exponentially by a power of 5, in order that the maximum asperity height is in excess of at least 3 standard deviations. By increasing the exponential power of the sequence up to 500, λ_*W*_-values up to 20 have been studied, though limitations of the random number generator start to yield numerical instabilities. For the purpose of establishing a trend line, as λ_*W*_-values over 3 are expected to yield negligibly small wear, the Monte Carlo study focused up to this asperity height.

**Fig 6:**
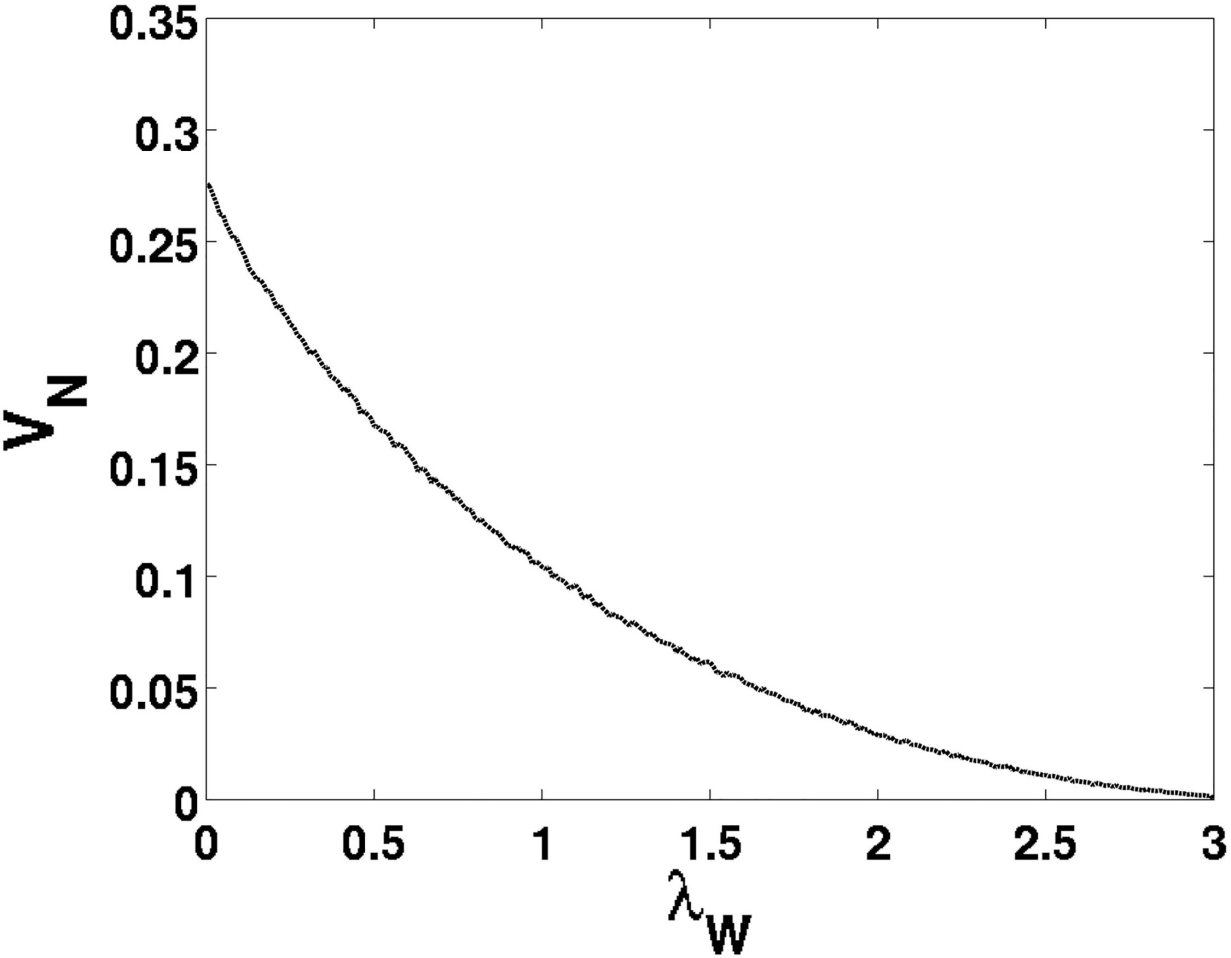
Monte Carlo data of *V*_*N*_ as a function of λ_*W*_.

For each λ_*W*_-value of interest, the unworn random sequence (Fig 5-a) is used and all asperities that exceed the given λ_*W*_-value (which represents the standard deviation ratio) were *worn*, where the height was reduced down to the λ_*W*_-value (Fig 5-b). The wear can be represented as

$$\begin{array}{ccc} V & = & \frac{\Delta x^{2}}{N}\sigma \Sigma_{i}^{N}\left(h_{i}-\lambda_{W}\right), \end{array}$$ (31)

where *h*_*i*_ represents the normalized (dimensionless) height of each random asperity, *N* is the total number of asperities studied in the Monte Carlo simulation, Δ*x*^2^ (m^2^) represents the area under contact, *σ* (m) represents the RMS surface roughness, and *V* (m^3^) is the total wear. For each asperity, the height worn off was collected and averaged throughout all of the asperities, to yield an average wear height relative to the area of contact. The numerically obtained ratio of normalized wear for a given λ_*W*_-value (Fig 6) comes out to

$$\begin{array}{ccc} V_{N} & = & 0.2763\cdot exp\left[-1.6754\cdot \lambda_{W}\right], \end{array}$$ (32)

and the dimensionless normalized wear volume *V*_*N*_ can apply for the given λ_*W*_-value regardless of the surface roughness or area of contact. The assumption that the wear rate follows an exponential function of the λ_*W*_-value has been well established [1].

To convert the normalized volume in Eq 32 to the real wear volume in Eq 31, one simply multiplies the normalized wear by the RMS surface roughness (asperity height) and the area of contact

$$\begin{array}{ccc} V & = & V_{N}\cdot \sigma \Delta x^{2}. \end{array}$$ (33)

**Fig 7:**
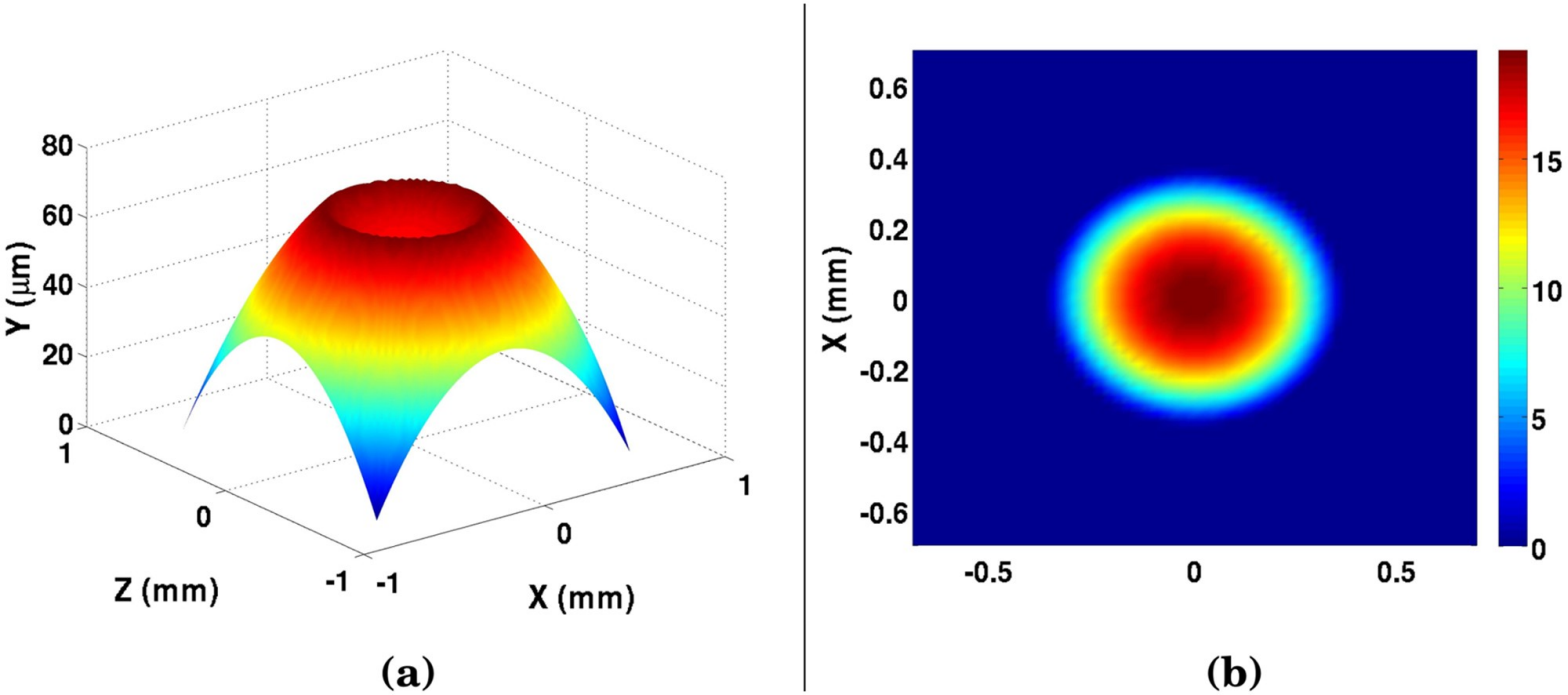
Numerical results of wear after 1 hour of sliding contact at a bulk temperature of 59°C and a load of 391 Newtons, both (a) with and (b) without the ball bearing profile. Colorbar in (b) represents wear in *μ*m.

This function assumes the total wear over a given area. In the four-ball test, however, the contact is transient, and therefore the wear rate is

$$\begin{array}{ccc} \dot{V} & = & V_{N}\cdot \sigma \Delta x\cdot U, \end{array}$$ (34)

where *U* (m/s) is the sliding speed, and $\dot{V}$ (m^3^/s) is the transient wear rate. By using this wear rate, and finding the λ_*W*_ obtained from the film thickness obtained with the pressure obtained by the Reynolds-function, as well as the minimum elastohydrodynamic film thickness (Eq 9), a transient wear profile can be obtained (Fig 7).

It is clear looking at this numerical method, as well as the initial ball bearing profiles in Eq 4, that the simulation is assuming a completely smooth ball bearing profile; in reality there are random asperities that are significant compared to the scale of the lubricant thickness, which could affect the results converged on with the iterative Reynolds solver. A more accurate simulation than that which is described with Eq 34 would have a bearing profile with asperities, and then directly determine whether an individual asperity exceeds the lubricant film thickness. This simulation, however, would have *dramatically* greater computational costs, as in order to truly get an accurate representation of random asperities, a parametric Monte Carlo simulation would need to be conducted at every condition, and with far greater resolution than the 61⋅61 resolution currently used. In addition, converging on a solution to the Reynolds equation with random asperities would be far longer and prone to errors in convergences. For the sake of computational efficiency, the wear rate equation defined in Eq 34 was used in this numerical simulation.

## Experimental procedure

**Fig 8:**
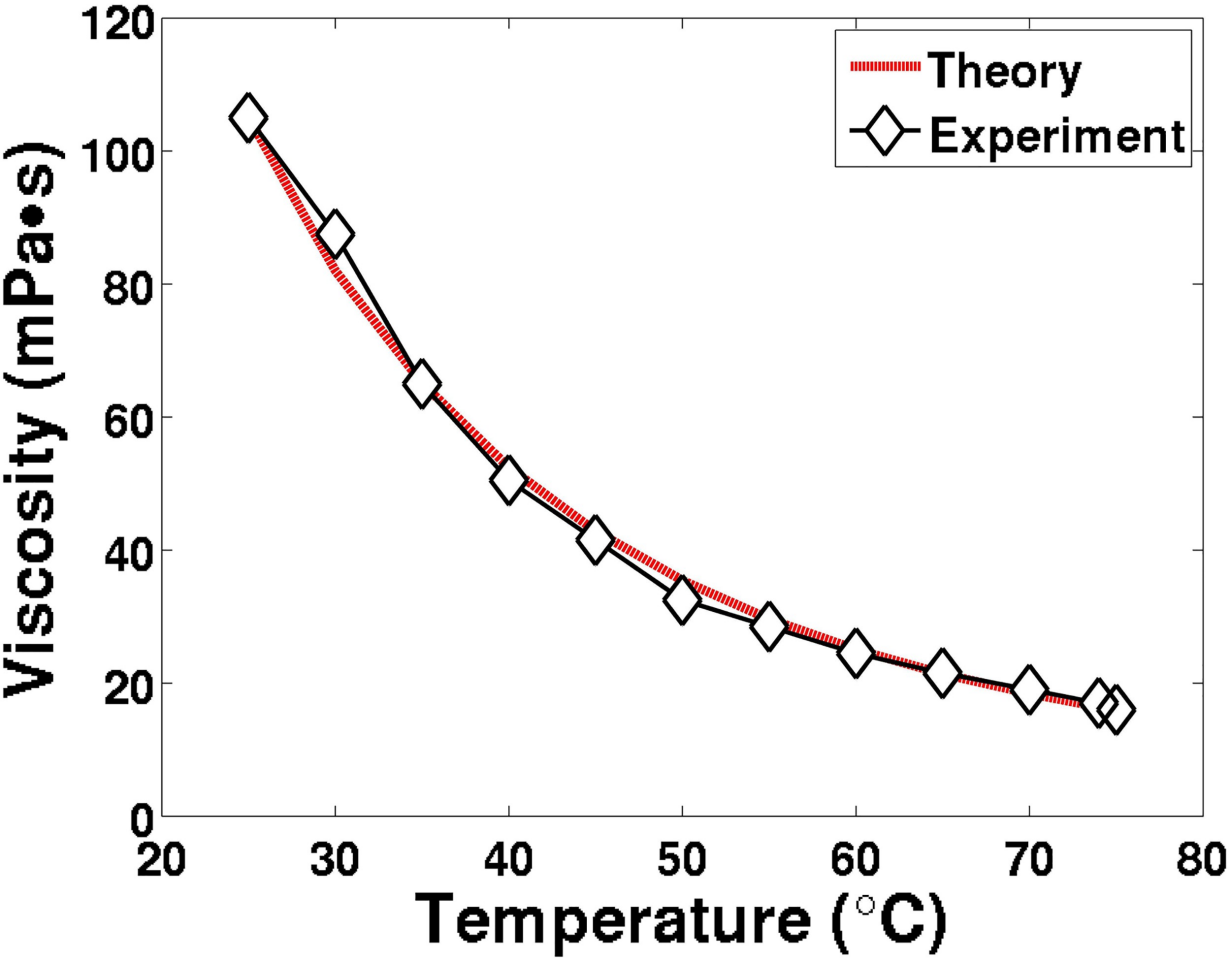
Mineral oil dynamic viscosity data.

A series of four-ball [56] sliding contact tests were conducted to experimentally characterize the wear over varying temperatures, loads, and lengths of time with mineral oil; the viscosity as a function of temperature was collected (Fig 8). The four-ball tests were set to consistently run at 1200 r/min, ramped up with an angular acceleration of 100 r/min per second. Throughout all of the tests, the angular force, and therefore the COF, was consistently recorded by a load cell within the four-ball apparatus. Three series of tests were conducted, the first in time variation, the second in load variation, and the third in temperature variation. For the first series of tests, the run time for each test was varied for different times to characterize the evolution of the wear; run-times used include 10, 60, 120, 300, 1800, and 3600 seconds after the test speed of 1200 r/min was reached. Throughout the time-variation experimental tests, the load was kept constant at 391 Newtons, and the oil was set at a consistent temperature of 51°C; Proportional-Integral-Derivative (PID) controllers and convection fans were used to maintain the temperature in the presence of flash heating. The second series of tests were all conducted at the full run-time of 3600 seconds, and a consistent temperature of 59°C, but with a variation of the load at 302, 347, and 391 Newtons. The third series of tests were all conducted at the full run-time of 3600 seconds and a load of 391 Newtons, but with a variation of the bulk oil temperature at 51°C, 59°C, and 67°C. Finally, every test was completed twice under identical circumstances, to ensure repeatability of the results.

## Results

After each four-ball test, all of the ball bearings were first cleaned in acetone and isopropyl alcohol, and then measured with an optical profilometer, which provides an accurate three-dimensional (3D) model of the wear scar on the ball bearing. The Metro-Pro MX software was utilized to mask the wear scar, and remove the material of the 0.25-inch radius sphere ball bearing. This sphere-removal algorithm enabled a true measurement of the total wear loss, with far greater accuracy than the traditional method of approximating wear loss based on the wear scar diameter.

**Fig 9:**
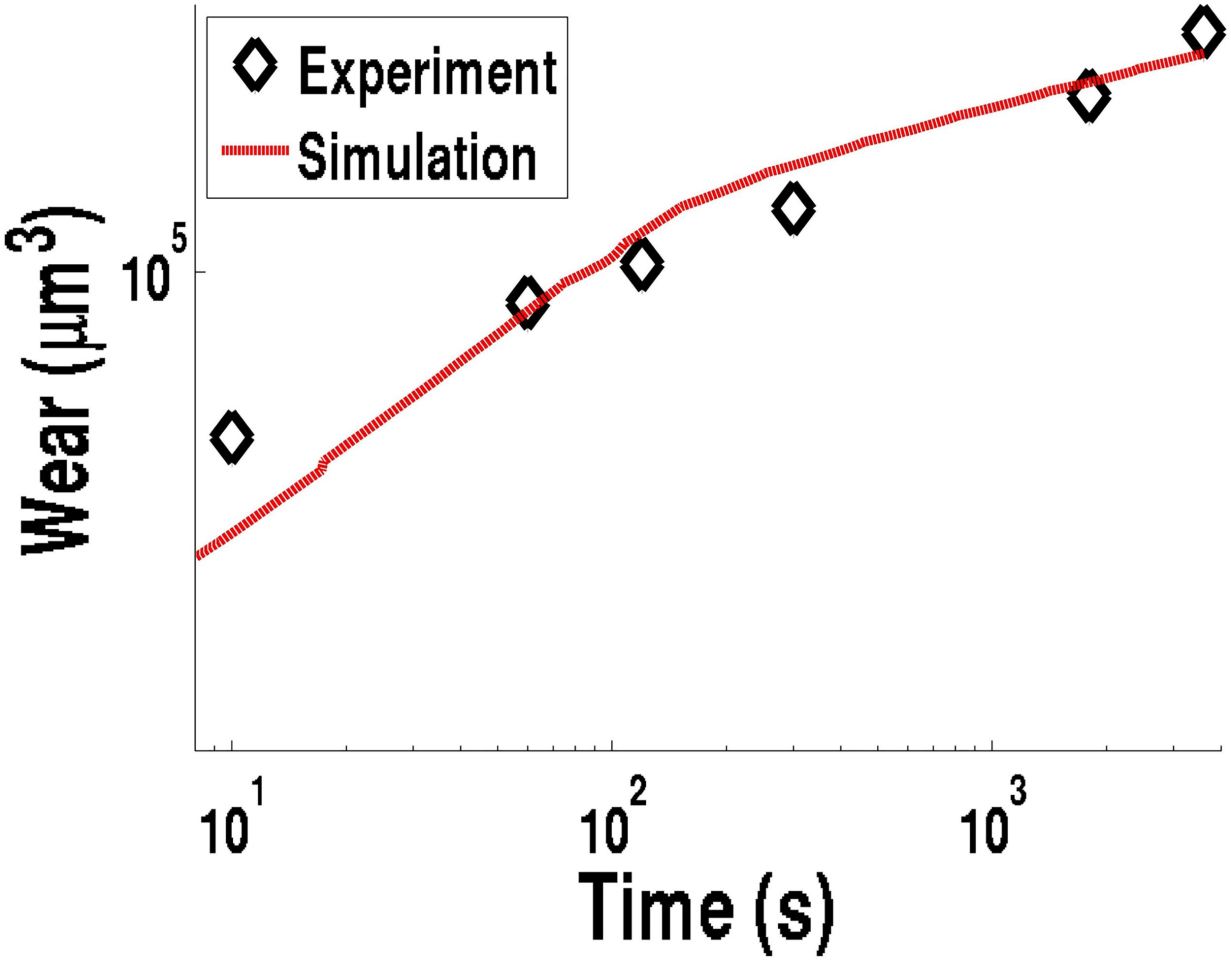
Wear (*μ*m^3^) experimental data and matching simulation results, for neat mineral oil at a bulk lubricant oil temperature of T = 51°C.

**Fig 10:**
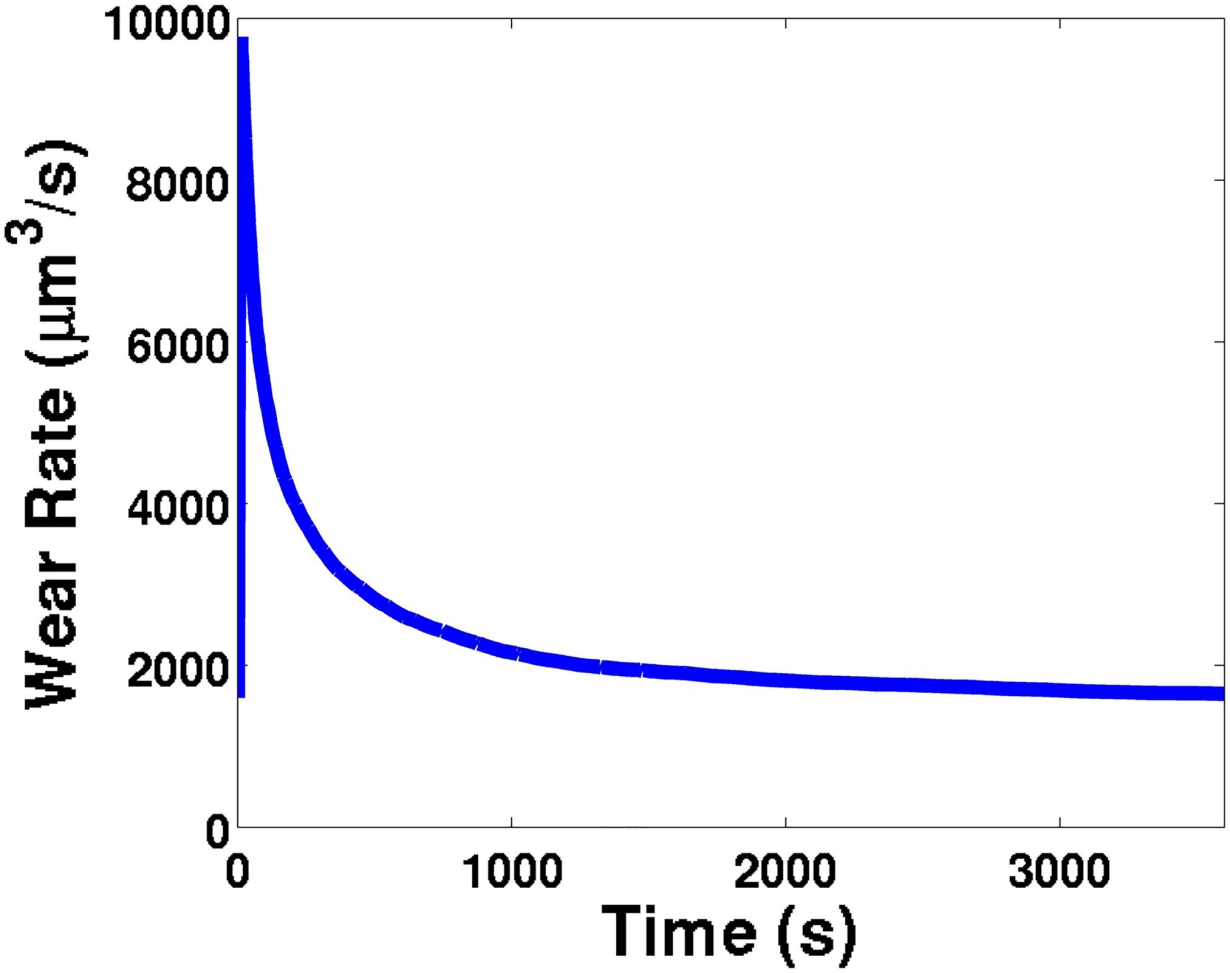
The phenomenon of running in, demonstrated from the numerical wear rate (*μ*m^3^/s) simulation results for neat mineral oil at a bulk lubricant temperature T = 59°C.

The total wear as a function of duration of the timed contact was collected at a constant load of 391 Newtons, and a consistent bulk lubricant oil temperature of 51°C (Fig 9). This data was compared to the numerically calculated wear, and the experimental data reflects the numerical results. The simulations show a gradual decrease in wear rate with increasing time and total wear (Fig 10); this is primarily caused by a reduction in friction heating density (Eq 26) due to the increase in contact area as the wear scar diameter increases. As the friction heating density decreases, the lubricant oil temperature decreases, which causes the viscosity and film thickness to increase, and thus gradually reducing the wear. This close match is further verification and validation of using this numerical approach as a reliable model of four-ball sliding contact tests, and strong evidence of the robustness of this model.

**Fig 11:**
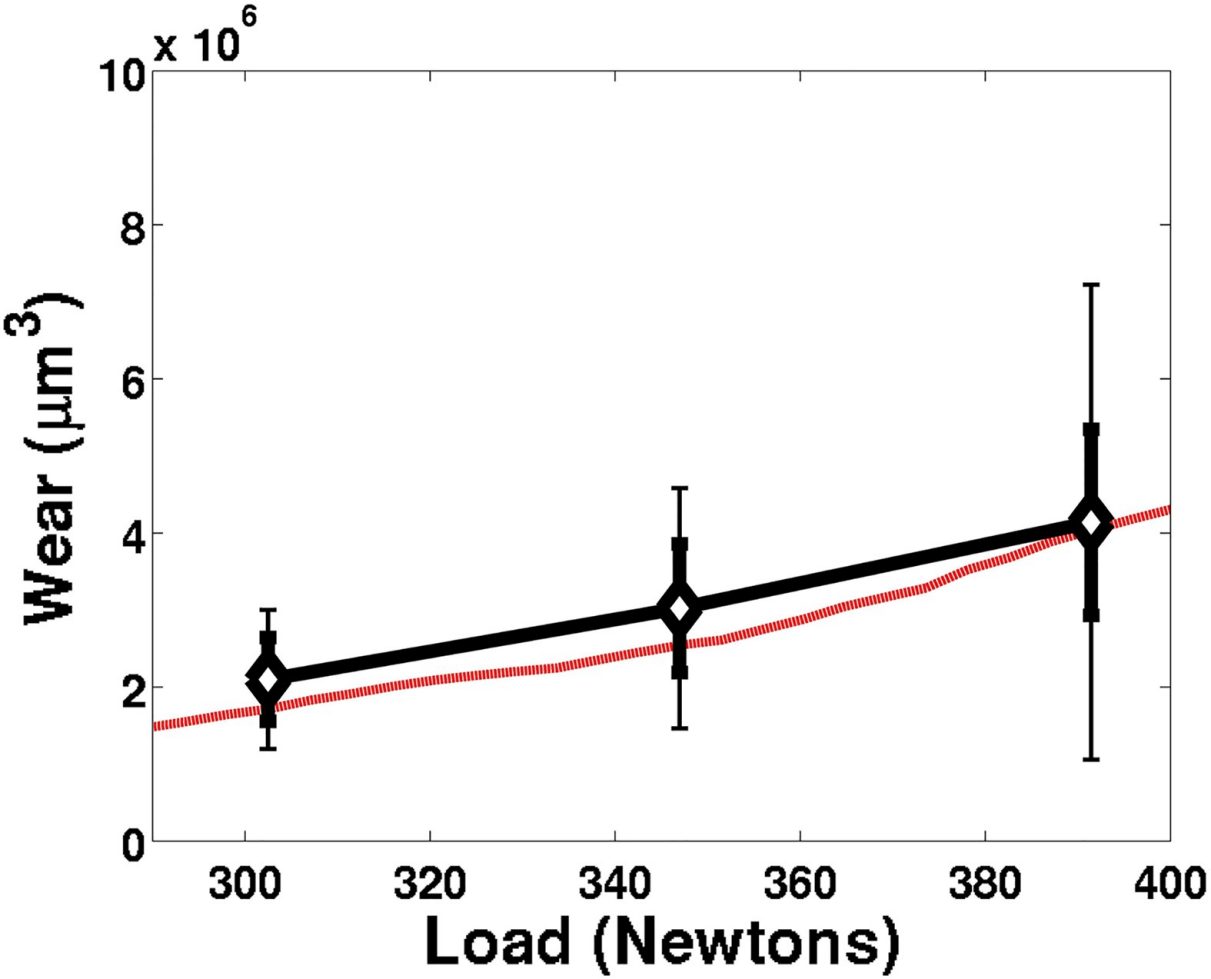
Wear (*μ*m^3^) experimental data and matching simulation results as a function of load (Newtons). Diamonds represent the experimental average total wear, while error bars represent the average (thick error bars) and maximum (thin error bars) experimental variation of the total wear observed between all six samples (two repeating tests with three ball bearings each).

Second, a series of 59°C, hour-long, four-ball tests were conducted at varying loads, ranging from 302 to 391 Newtons. It is expected that, with all other parameters consistent, as the load increases, the wear rate will increase, as noticed in Archard’s Eq 1 and Hamrock-Dowson Eqs 9 and 10. All of the simulation-predicted wear volumes (Fig 11) reasonably match the experimental load-dependent wear rates, and a clear trend of increasing wear with increasing load is observed both numerically and experimentally.

**Fig 12:**
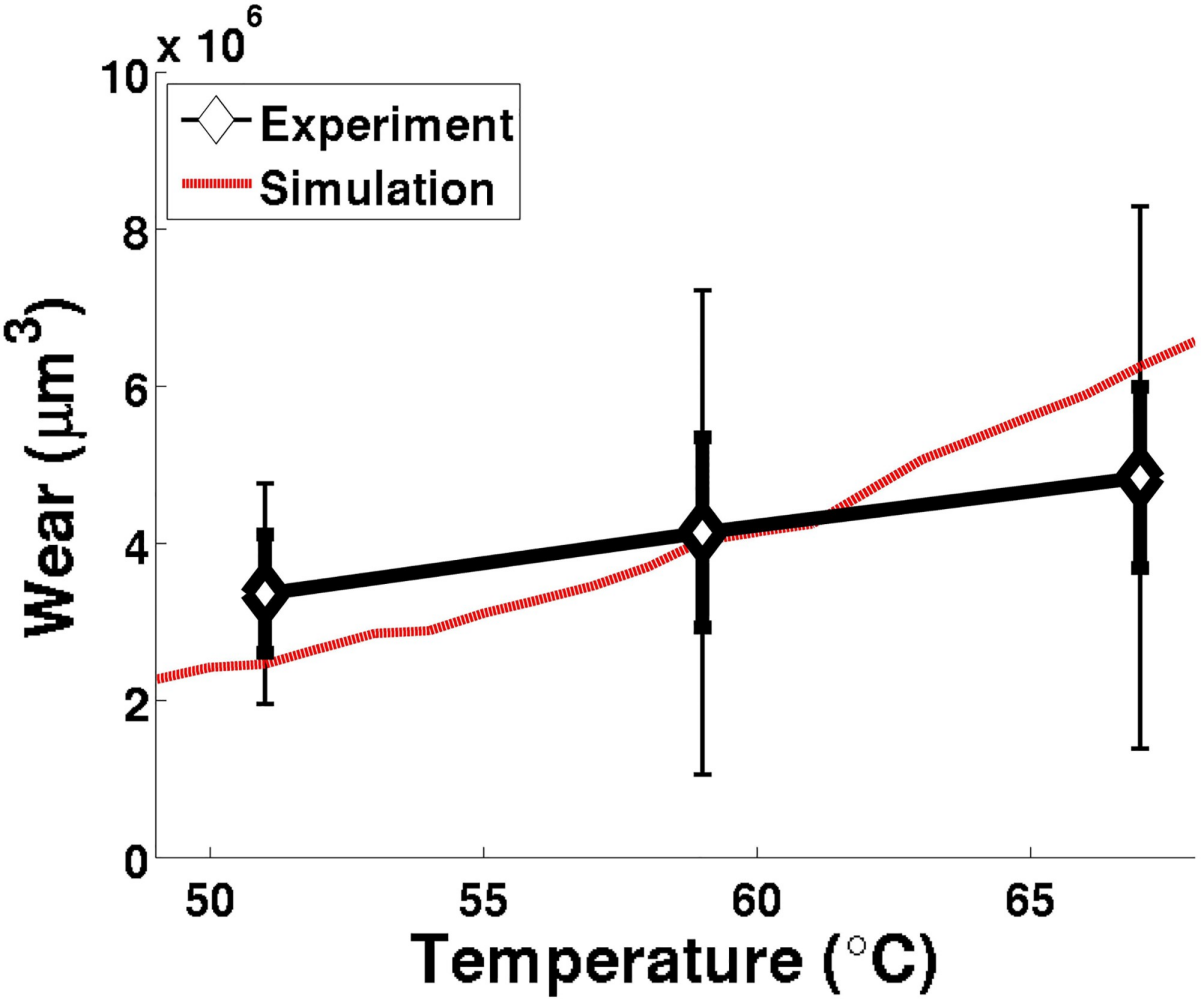
Wear (*μ*m^3^) experimental data and matching simulation results as a function of bulk lubricant oil temperature (°C). Diamonds represent the experimental average wear, while error bars represent the average (thick error bars) and maximum (thin error bars) experimental variation of the wear observed between all six samples (two repeating tests with three ball bearings each).

Finally, a series of hour-long, 391 Newton load, four-ball tests were conducted at varying bulk temperatures, ranging from 51°C to 67°C. It is expected that, with all other parameters consistent, as the bulk temperature increases, the wear volume will increase. The higher temperatures oils will inherently have a reduced viscosity, and a reduction in viscosity will result in a decrease in minimum and central lubricating oil thickness, as noticed in Eqs 9 and 10. This trend is observed both experimentally and numerically, and the simulation-predicted wear volumes (Fig 12) reflected the experimental data. This match helps to further establish this model as a robust representation of sliding contact within a four-ball test.

## Conclusion

A novel numerical model was developed using established elastohydrodynamic principles. The numerical model used a series of iterations at each time-step in order to successfully converge at an accurate prediction of the pressure distribution, elastic deflection, lubricant film thickness, lubricant temperature, and lubricant viscosity. A Reynolds equation solver was developed to determine the pressure distribution, in conjunction with the Roelands equation to find the viscosity increase with pressure. The Winkler Mattress model was used to predict the elastic deformation of the ball-bearing surface as a result of pressure, and the Hamrock-Dowson empirical equation was used to determine the minimum elastohydrodynamic film thickness at the edge of the contact. Finally, a Monte-Carlo simulation was conducted to predict the wear rate as a result of the ratio of RMS surface roughness over the lubricant oil film thickness, and an empirical exponential equation was obtained from this numerical study.

A series of four-ball sliding contact tests were conducted to validate this numerical model. The simulated wear predictions reasonably matched experimental trends resulting from variations in time, load, and temperature. Over time, the total wear consistently increased, though the average wear rate would decrease with increasing total wear, primarily due to the decreased friction heating density at the enlarged area of contact. The wear was observed both experimentally and numerically to increase with increasing load, as expected based on Archard’s Wear Equation. Finally, as the temperature increased, the viscosity and thus lubricant film thickness would decrease, resulting in an increase in wear; this was observed both numerically and experimentally. With this experimentally validated numerical model, an engineer can substitute extensive parametric four-ball sliding contact tests, which require expensive equipment and significant amounts of time, with cheap and straightforward parametric simulations; this will reduce the need for excessive experiments and improve overall engineering design.

## Supporting information

S1 File A self contained PDF document that contains a list of symbols, all of the MATLAB source code with embedded program flow charts, a manual for the code’s operation, and tabulated experimental data. (PDF)
