# Supplementary material for: Tribological investigations of the load, temperature, and time dependence of wear in sliding contact
Source: PLoS One. 2017 Apr 20;12(4):e0175198. doi: 10.1371/journal.pone.0175198 (PMC5398515; doi:10.1371/journal.pone.0175198)
Supplement: S1 File — (PDF) [file pone.0175198.s001.pdf]

Symbols

- V (m^3), total wear
- $\dot{V}$ (m^3/s), wear rate
- P (Pa), pressure
- S (m), total distance of sliding contact
- U (m/s), velocity of sliding contact
- σ (m), RMS surface roughness
- h (m), oil thickness
- F_{indent} (m), the profile function of the ball bearing
- V_y (m), wear profile depth
- δ_e (m), elastic deflection of ball bearing
- h_{min} (m), minimum lubricant thickness
- U_n, dimensionless speed parameter
- G_n, dimensionless material parameter
- W_n, dimensionless load parameter
- R (m), radius of ball bearing
- K_h (Pa/m), Winkler Mattress Coefficient
- E (Pa), Young's Modulus
- p, Poisson's Ratio
- R' (m), reduced radius
- E' (Pa), reduced Young's modulus
- a_{Hertz} (m), radius of Hertzian elastic contact area
- a (m), radius of contact area
- P_{Hertz} (Pa), Hertzian pressure

- δ_{Hertz} (m), Hertzian deflection
- *x*, *y*, and z (m), dimensions
- *r* (m), radial distance from a given point to the center
- Δx (m), the distance increment of each finite-difference node
- ν_0 (mm^2/s), kinematic viscosity at atmospheric pressure
- ν_P (mm^2/s), kinematic viscosity under high contact pressure
- μ (Pa-s), dynamic viscosity
- *L*, Peclet number
- $\kappa_{ellipse}$, the wear scar ellipticity
- α_{bb} (m^2/s), thermal diffusivity of ball bearing material
- α_{lub} (m^2/s), thermal diffusivity of lubricant oil
- k_{bb} ($\mathrm{W/m{\cdot}^\circ C}$), thermal conductivity of ball bearing material
- k_{lub} ($\mathrm{W/m{\cdot}^\circ C}$), thermal conductivity of lubricant oil
- $C_{P,bb}$ ($\mathrm{J/kg{\cdot}^\circ C}$), specific heat of ball bearing material
- $C_{P,lub}$ ($\mathrm{J/kg{\cdot}^\circ C}$), specific heat of lubricant oil
- ρ_{bb} (kg/m^3), density of ball bearing material
- ρ_{lub} (kg/m^3), density of lubricant oil
- Ω_{RPM} (r/min), rotational speed of four-ball test
- *W* (Newtons), load
- μ_{COF}, Coefficient of Friction
- ΔT_F (°C), friction temperature increase at the surface
- t_{ss} (s), time for flash temperature heating to settle
- $T_{surface}$ (°C), the temperature at the surfaces of the ball bearings
- T_B (°C), the bulk lubricant oil temperature

- $T_L(y)$ (°C), temperature profile of the lubricant film
- T_L (°C), average temperature of the lubricant film
- Q_{lub} (Watts), power from friction forces into lubricant film
- V_{lub} (m^3), volume of lubricant over area of contact
- q_v (Watts/m^3), power per unit volume into lubricant
- h_c (m), film thickness where *dP/dx* = 0
- U_x and U_z (m/s), flow in *x* and *z* direction,
- γ (m), length of individual asperities
- $\bar{\gamma}$ (m), mean value of all asperities within the wear scar
- N_a, number of finite difference points within the wear scar
- W_P (m), yield / plasticity length
- G_{yield} (Pa), shear yield strength of ball-bearing material
- λ_W, ratio of wear height over RMS asperities
- V_N, normalized wear rate
- *N*, total number of Monte Carlo trials performed ($N = 10^9$)
- *H* (Pa), ball bearing material hardness
- *f*, definition of a function
- θ (radians), trigonometric angle for ball-bearing indentation function
- (*m*), the dimension of length, typically meters
- (*Pa*), the dimension of pressure, typically Pascals

MATLAB SOURCE CODE

WearStudy.m

```
clear
tic

Tb=59;            % Bulk Lubricant Temperature (Centigrade)
maxdepth=5e-8;    % Maximum depth of wear per cycle
maxdt=1e0;        % Maximum time step per cycle
Mx=201;           % X and Z Nodes
W0=88;            % Applied load in lbs for four ball tester
Ra0=15.0e-8;      % Initial surface roughness of ball bearing
nd=0e-4;          % weight fraction of diamond nanoparticles



totaltime=3600;   % Total Wear Time (seconds)
Ey=210e9;         % Young's Modulus of the tested material in Pa
poisson=.3;       % Poisson Ratio of tested material
R = 0.25;         % Radius of ball bearings in inches
RPM=1200;         % Speed of spindle in rpm
COF=0.10;         % COF of interest
dt0=0e0;          % Initial starting time step (s)
Bw=0.0014;        % Bearing width (meters)
UTC=5e8;          % Ultimate tensile stress of steel (Pa)
SC=0.6;           % Ultimate shear stress coefficient for steel
Ea=-41e-21;       % Diamond Nanoparticle Activation Energy (J)
K0=900;           % Therman Conductivity Constant
k_boltz=1.38e-23; % Boltzman Constant
```

```
k=46.6;           % Thermal Conductivity of Material (W/m-K) - Steel
rho=7810;         % Material Density (kg/m^3) - Steel
Cp=475;           % Specific Heat of Material (J/kg-K) - Steel

k_d=2190;         % Thermal Conductivity of Material (W/m-K) - Diamond
rho_d=3530;       % Material Density (kg/m^3) - Diamond
Cp_d=519;         % Specific Heat of Material (J/kg-K) - Diamond

k_oil=0.140;      % thermal conductivity of oil (W/m-K)
Cp_oil=2000;      % specific heat of oil (J/kg-K)
rho_oil=905.75;   % Density of Lubricant (kg/m^3)

%%%%%%%%%%%%%%%%%%

if mod(Mx,2)==0
    Mx=Mx+1;
end
dx=Bw/Mx;
Xfct=linspace(-Bw/2,Bw/2,Mx);
W=W0*4.44822162/3;  % Divide by 3 and convert to Newtons
R=R*2.54/100;
Rp=R/2;
Eyr=Ey/(1-(poisson^2));
aHertz=((3/2)*W*Rp/Eyr)^(1/3);
omg=RPM*(2*pi/60);
U=0.5*omg*R; % Contact Speed (m/s)
ndx=nd*(1e4);
k_nd=(1+(K0*exp(Ea/(k_boltz*(Tb+273.15)))))*k_oil;
k_lub=(k_oil*(1-ndx))+(k_nd*ndx);
Cp_lub=(Cp_oil*(1-nd))+(Cp_d*nd);
rho_lub=1/(((1-nd)/rho_oil)+(nd/rho_d));
TD=k/(rho*Cp); % Thermal Diffusivity (m2/s)
TD_lub=k_lub/(rho_lub*Cp_lub); % Thermal Diffusivity of oil (m2/s)
kellip=1;                % Elliptical Parameter = a/b
oo=ceil(Mx/2);
Wp=Rp*((UTC*SC/Eyr)^2);
PmaxH=((1.5*W)/(pi*(aHertz^2)));
delH=1.31*(((W^2)/((Eyr^2)*Rp))^(1/3));
Kh=4*PmaxH/delH;
Rw=Rp*((2*pi*R/(0.5*Bw/4.5)));
```

```
% Viscosity
VcSt0=ViscFct(Tb);
b0=0.6363/0.2; % Taken
PVC=(1.216 + (4.143*((log10(VcSt0))^3.0627))+...
((2.848e-4)*(b0^5.1903)*((log10(VcSt0))^1.5976))...
-(3.999*((log10(VcSt0))^3.0975)*((rho_lub/1000)^0.1162)))*(1e-8);

% Calculate initial film thickness
Vpas0=VcSt0*rho_lub*(1e-6);
Vpas=Vpas0;

%%%%%%%%%%%%%%%%%%%

a=aHertz;
L=U*a/(2*TD); % Peclet Number (dimensionless)
if L<0.1
    Tf=0.25*COF*W*U/(k*a);
elseif L>0.1 && L<5
    foo=0.35+((5-L)*(0.5/4.9));
    Tf=foo*0.25*COF*W*U/(k*a);
else
    Tf=(0.308*COF*W*U/(k*a))*(sqrt(TD/(U*a)));
end

foo=0:0.001:1; foo=foo-(foo.^2); foo=mean(foo);
HcHD=Rw*2.69*((U*Vpas/(Eyr*Rp))^0.67)*((PVC*Eyr)^0.53)*...
((W/(Eyr*(Rp^2)))^-0.067)*(1-(0.061*exp(-0.73*kellip)));
HcHD0=HcHD; Fluc=1; ct=0;
while Fluc>(1e-2)
    ct=ct+1;
    dT=foo*COF*(W/(pi*(aHertz^2)))*HcHD*U/(2*k_lub); Ti=Tb+Tf+dT;
    VcSt=ViscFct(Ti); Vpas=VcSt*rho_lub*(1e-6);
    HcHD=Rw*2.69*((U*Vpas/(Eyr*Rp))^0.67)*((PVC*Eyr)^0.53)*...
    ((W/(Eyr*(Rp^2)))^-0.067)*(1-(0.061*exp(-0.73*kellip)));
    Fluc=abs(HcHD-HcHD0)/HcHD0;
    HcHD0=HcHD;
    FlucFct(ct)=Fluc;
    if ct>10000
        Fluc=0;
```

```
        ['Trouble!!!']
    end
end

H0HD=Rw*3.63*((U*Vpas/(Eyr*Rp))^0.68)*((PVC*Eyr)^0.49)*...
((W/(Eyr*(Rp^2)))^-0.073)*(1-(exp(-0.68*kellip)));

wear=zeros(Mx,Mx);


% Indent Function
indent=zeros(Mx,Mx);
for ii=1:Mx
    for jj=1:Mx
        rrp=sqrt((Xfct(ii)^2)+(Xfct(jj)^2));
        theta=asin(rrp/R);
        indent(ii,jj)=R*(1-(cos(theta)));
    end
end
Rfct=zeros(Mx,Mx);
for ii=1:Mx
    for jj=1:Mx
        Rfct(ii,jj)=sqrt((Xfct(ii)^2)+(Xfct(jj)^2));
    end
end

%%%%%%%%%%%%%%%%%

% Find a default pressure function to start iteration
[aa,bb]=find(Rfct<aHertz); Laa=length(aa);
PfctH=zeros(Mx,Mx);
for uu=1:Laa
    ii=aa(uu); jj=bb(uu);
    PfctH(ii,jj)=PmaxH*(1-((Rfct(ii,jj)/aHertz)^2));
end


[h0,Pfct00]=gethfct(indent,wear,dx,Vpas,Vpas0,H0HD,U,...
PfctH,R,Kh,PVC,Tb,Ti);
```

```
h=h0; havg=h;
Pfct0=Pfct00; Pfct=Pfct00;

a=aHertz;
time=0;
dTavg=0;
NL=['\n'];
TS=0;

dt=dt0;
while (time<totaltime)
    TS=TS+1;
    time=time+dt;

    [a0,kellip]=wearscar(wear,dx,Ra0/2);
    aD=a0*2; a=a0;
    if (a<aHertz)
        a=aHertz;
    end

    %%%%%%%%%%%%%%%%%%%%%%%%%

    L=U*a/(2*TD); % Peclet Number (dimensionless)
    if L<0.1
        Tf=0.25*COF*W*U/(k*a);
    elseif L>0.1 && L<5
        foo=0.35+((5-L)*(0.5/4.9));
        Tf=foo*0.25*COF*W*U/(k*a);
    else
        Tf=(0.308*COF*W*U/(k*a))*(sqrt(TD/(U*a)));
    end

    foo=0:0.001:1; foo=foo-(foo.^2); foo=mean(foo);
    HcHD=Rw*2.69*((U*Vpas/(Eyr*Rp))^0.67)*((PVC*Eyr)^0.53)*...
    ((W/(Eyr*(Rp^2)))^-0.067)*(1-(0.061*exp(-0.73*kellip)));
    HcHD0=HcHD; Fluc=1; ct=0;
    while Fluc>(1e-2)
        ct=ct+1;
        dT=foo*COF*(W/(pi*(aHertz^2)))*HcHD*U/(2*k_lub); Ti=Tb+Tf+dT;
        VcSt=ViscFct(Ti); Vpas=VcSt*rho_lub*(1e-6);
```

```
    HcHD=Rw*2.69*((U*Vpas/(Eyr*Rp))^0.67)*((PVC*Eyr)^0.53)*...
    ((W/(Eyr*(Rp^2)))^-0.067)*(1-(0.061*exp(-0.73*kellip)));
    Fluc=abs(HcHD-HcHD0)/HcHD0;
    HcHD0=HcHD;
    FlucFct(ct)=Fluc;



    if ct>10000
        Fluc=0;
        ['Trouble!!!']
    end
end

H0HD=Rw*3.63*((U*Vpas/(Eyr*Rp))^0.68)*((PVC*Eyr)^0.49)*...
((W/(Eyr*(Rp^2)))^-0.073)*(1-(exp(-0.68*kellip)));

%%%%%%%%%%%%%%%%%%%%%%%%%%%%%

[aa,bb]=find(Rfct<(a-dx)); Laa=length(aa);

PfctHt=zeros(Mx,Mx);
for uu=1:Laa
    ii=aa(uu); jj=bb(uu);
    PfctHt(ii,jj)=((1.5*W)/(pi*(a^2)))*(1-((Rfct(ii,jj)/a)^2));
end

[h,Pfct]=gethfct(indent,wear,dx,Vpas,Vpas0,H0HD,U,Pfct00,R,...
Kh,PVC,Tb,Ti);

[aa,bb]=find(Rfct<a); Laa=length(aa);



RaOP=Ra0; MW=(mean(mean(wear(aa,bb))));
for uu=1:Laa
    ii=aa(uu); jj=bb(uu);
    RaOP=RaOP+((wear(ii,jj)-MW)^2);
end
RaOP=2*sqrt((RaOP/Laa)+(Ra0^2));
```

```
RaExp=((0.0027125069*time) + (0.8618890914))*(1e-6);

Ra=Ra0;

LamFct=(h+Wp).*(Ra.^-1);
LamRat=0.2763*exp(-(1.6754)*LamFct);
wearrate=(LamRat.*Ra.*(U*dx))/(dx^2);
for ii=1:Mx
    for jj=1:Mx
        if wearrate(ii,jj)<0
            wearrate(ii,jj)=0;
        end
    end
end
% The wear rate is in m/s in this model
WRt=(sum(sum(wearrate)))*(dx^2)*(1e18);
WRavg=(sum(sum(wear)))*(dx^2)*(1e18)/time;



maxwearrate=max(max(wearrate));
if maxwearrate>0
    dt=maxdepth/maxwearrate;
else
    dt=totaltime;
end
if (dt>maxdt)
    if (mod(time,maxdt)~=0)
        dt=maxdt-(mod(time,maxdt));
    else
        dt=maxdt;
    end
end
wear=wear+(wearrate*dt);

COFrat=0.5*exp(-1.2847*LamFct);
F_wear=UTC*SC*(sum(sum(COFrat)))*(Bw^2)/3;
COFw=F_wear/W;

WearData(TS)=(dx^2)*sum(sum(wear));
```

```
    WearRateData(TS)=(dx^2)*sum(sum(wear))/time;
    WearRateSimData(TS)=(dx^2)*sum(sum(wearrate));
    COFdata(TS)=COFw;
    aData(TS)=aD;
    kellipData(TS)=kellip;
    TimeData(TS)=time;
    RaData(TS)=mean(mean(Ra));
    RaOPdata(TS)=RaOP;
    RaExpData(TS)=RaExp;
    PressureMaxData(TS)=max(max(Pfct));
    PressureAvgData(TS)=mean(mean(Pfct));
    PressureScarData(TS)=mean(mean(Pfct(aa,bb)));
    hminData(TS)=H0HD;
    TempData(TS)=Ti;
    LamData(TS)=mean(mean(LamFct(aa,bb)));
    LamDataMax(TS)=max(max(erfc(LamFct(aa,bb))));
    hData(TS)=mean(mean(h(aa,bb)));
end

if nd==0
    filestr=['DataT' num2str(Tb) '.mat'];
else
    ndx=(1e-4)/nd;
    filestr=['DataT' num2str(Tb) 'd' num2str(ndx) '.mat'];
end

save(filestr)
toc
```

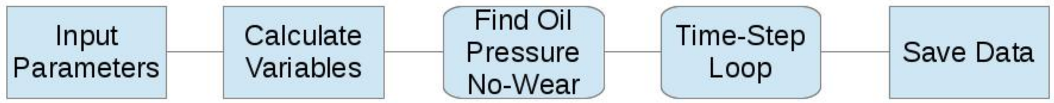

Figure 1: Highest level flowchart of four-ball test wear model. The process takes place within the *WearStudy.m* script.

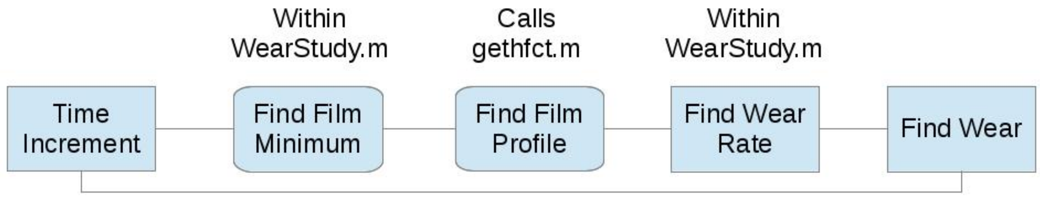

Figure 2: Flowchart representation of a single time-step. The process takes place within the *WearStudy.m* script.

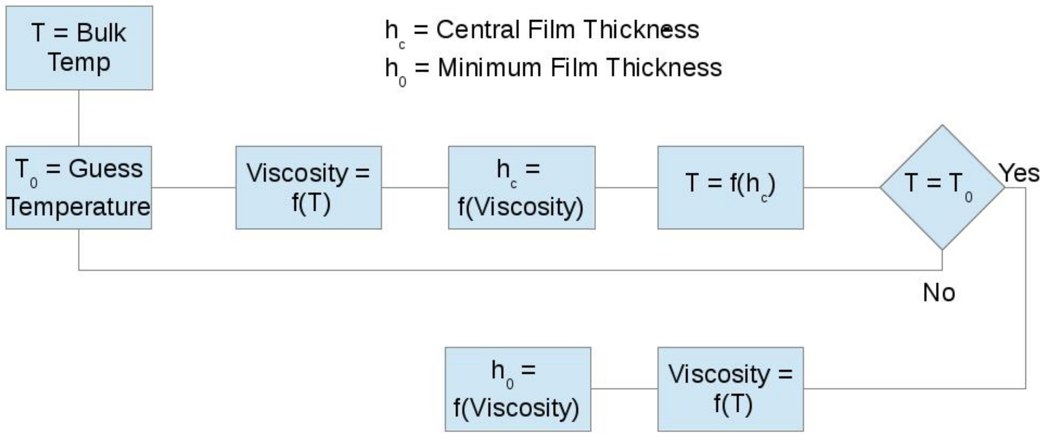

Figure 3: Flowchart representation of the iterative process of determining the lubricant film temperature, viscosity, and minimum thickness. The process takes place within the *WearStudy.m* script.

gethfct.m

```
function [hOut,PfctOut]=gethfct(indent,wear,dx,Vpas,Vpas0,...
H0HD,U,Pfct00,R,Kh,PVC,Tb,Ti)

Pfct0=Pfct00;

Mx=length(Pfct00); %oo=ceil(Mx/2);
ElastFct=zeros(Mx,Mx);

wear2=zeros(Mx,Mx);
for ii=1:Mx
    foo=(sum(wear(ii,:)))*(dx/(pi*R));
    for jj=1:Mx
        wear2(ii,jj)=foo;
    end
end

testbreak=0; del=1;
while del>1e-1
    testbreak=testbreak+1;
    ElastFct0=ElastFct;
    hFoo=wear+wear2+(indent*2)+ElastFct;
    h=hFoo-(min(min(hFoo)))+H0HD;

    [Pfct]=findP(h,Mx,dx,Vpas,Vpas0,Tb,Ti,U,PVC,Pfct0);
    ElastFct=Pfct/(Kh);

    del=abs(mean(mean(ElastFct-ElastFct0)))/(mean(mean(ElastFct)));
    Pfct0=Pfct;
    if testbreak<5
        del=1;
    elseif testbreak>25
        del=0;
        fprintf('Failure to Converge!!!!!!!');
    end
end

hFoo=wear+wear2+(indent*2)+ElastFct;
```

```
h=hFoo-(min(min(hFoo)))+H0HD;

hOut=h;
PfctOut=Pfct;

end
```

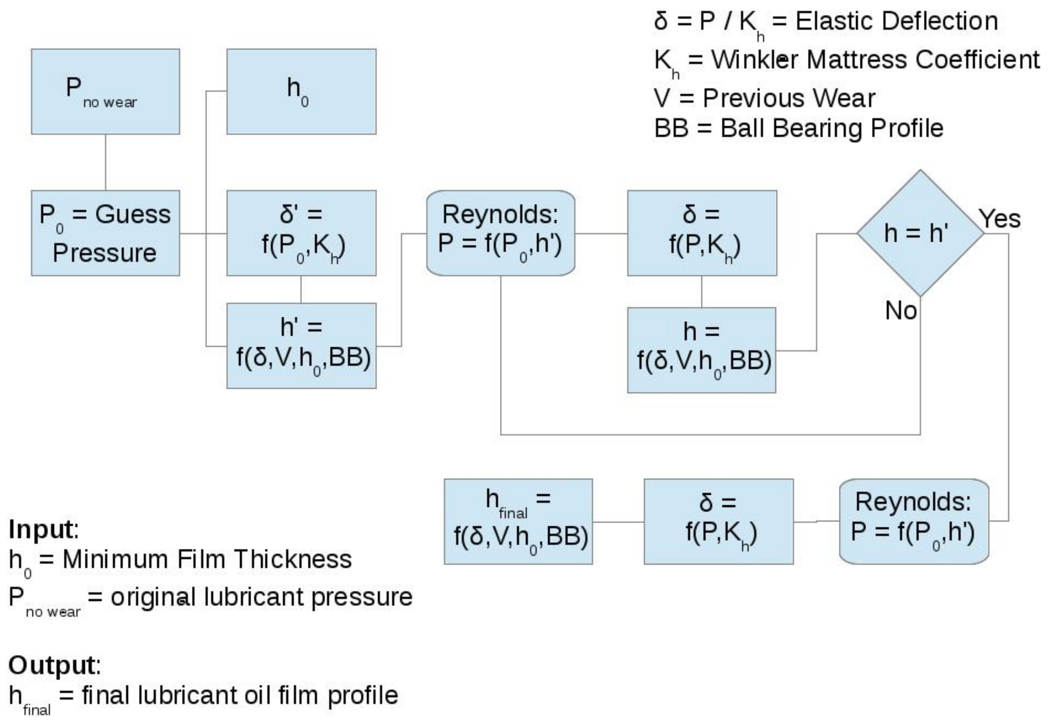

Figure 4: Flowchart representation of the determination of the lubricant film-thickness profile, which is performed within the *gethfct.m* function.

findP.m

```
function [PfctOut]=findP(h,Mx,dx,Vpas,Vpas0,Tb,Ti,U,PVC,Pfct0)

Pfct=Pfct0; DifMat=zeros(Mx,Mx,5);
AP=Roelands(Tb,Ti,PVC,Vpas,Vpas0,Pfct0);

for ii=2:(Mx-1)
    for jj=2:(Mx-1)
        uu1=(h(ii,jj)^3)/(dx^2);
        uuX=((1.5/dx)*(h(ii,jj)^2)*((h(ii+1,jj)-h(ii-1,jj))/(2*dx)));
        uuY=((1.5/dx)*(h(ii,jj)^2)*((h(ii,jj+1)-h(ii,jj-1))/(2*dx)));
        DifMat(ii,jj,1)=-((2/(dx^2))*(h(ii,jj)^3))-((2/(dx^2))*...
        (h(ii,jj)^3));              % A
        DifMat(ii,jj,2)=uu1+uuX;   % E
        DifMat(ii,jj,3)=uu1-uuX;   % W
        DifMat(ii,jj,4)=uu1+uuY;   % N
        DifMat(ii,jj,5)=uu1-uuY;   % S
    end
end




for ii=2:(Mx-1)
    for jj=2:(Mx-1)
        BB=6*Vpas*U*(((h(ii+1,jj)-h(ii-1,jj))/(2*dx))+...
        ((h(ii,jj+1)-h(ii,jj-1))/(2*dx)))*0.5;
        BB=BB*(exp(AP(ii,jj)));
        foo=(DifMat(ii,jj,2)*Pfct0(ii-1,jj));
        foo=foo+(DifMat(ii,jj,3)*Pfct0(ii+1,jj));
        foo=foo+(DifMat(ii,jj,4)*Pfct0(ii,jj-1));
        foo=foo+(DifMat(ii,jj,5)*Pfct0(ii,jj+1));
        Pfct(ii,jj)=(BB-foo)/(DifMat(ii,jj,1));
    end
end

PfctOut=abs(Pfct);
```

```
end
```

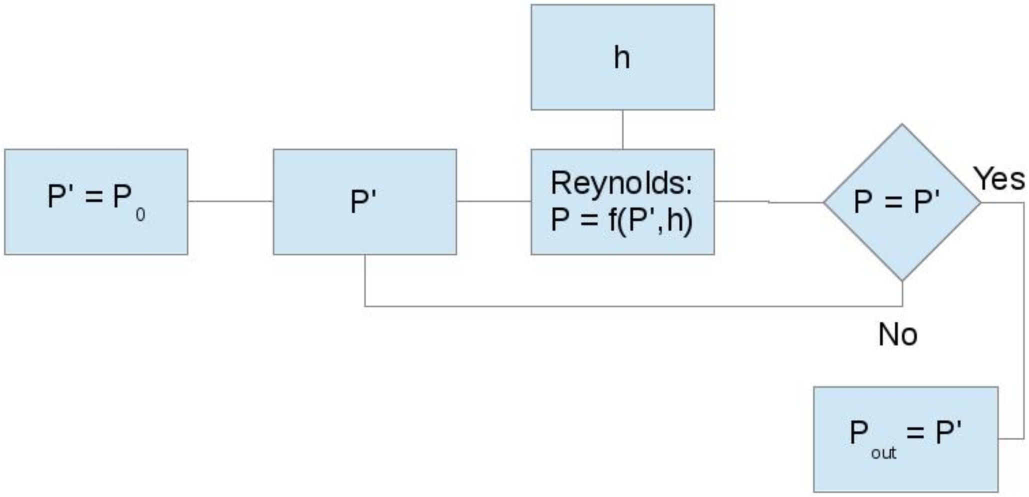

Figure 5: Flowchart representation of the determination of the lubricant pressure profile with the Reynolds equation, which is performed within the *findP.m* function.

Roelands.m

```
function [AP]=Roelands(TbC,TiC,PVC,Vpas,Vpas0,Pfct)

Ti=TiC+273.15; Tb=TbC+273.15;

foo=((log(Vpas))+9.67);
foo0=((log(Vpas0))+9.67);

Z=PVC/((5.1e-9)*foo0); %Z=0.68;
beta=GetBeta;
S0=beta*(Tb-138)/foo;
AP=(((Ti-138)/(Tb-138)).^-S0).*(((1+(5.1e-9*Pfct)).^Z)-1)*foo;

end
```

GetBeta.m

```
function [beta]=GetBeta

Tfct=25:5:75; LL=length(Tfct);
ViscDat=[105 87.5 65 50.5 41.5 32.5 28.5 24.5 21.5 19 16];

ViscRat=ViscDat(2:LL)/ViscDat(1);
ViscLog=log(ViscRat);
dTemp=Tfct(2:LL)-Tfct(1);

BetaFct=-ViscLog.*(dTemp.^-1);

beta=mean(BetaFct);

end
```

ViscFct.m

```
function [VcSt]=ViscFct(Tc)

T=Tc+273.15;
Tfct=25:5:75;
ViscDat=[105 87.5 65 50.5 41.5 32.5 28.5 24.5 21.5 19 16];

Tfct=Tfct+273.15;
foo=abs(T-Tfct);
a=find(foo==min(foo)); a=a(1);

if min(foo)==0
    b=a;
else
    if a==1
        b=2;
    elseif a==length(Tfct)
        b=length(Tfct)-1;
    else
        fooT=((T-Tfct(1))/5)+1;
        a=floor(fooT);
        b=ceil(fooT);
    end
end

if a==b
    VcSt=ViscDat(a);
else
    T1=Tfct(a); v1=ViscDat(a);
    T2=Tfct(b); v2=ViscDat(b);
    Z1=v1 + 0.7 + exp((-1.47-(1.84*v1)-(0.51*(v1^2))));
    Z2=v2 + 0.7 + exp((-1.47-(1.84*v2)-(0.51*(v2^2))));
    B=((log10(log10(Z1)))-(log10(log10(Z2))))/...
    ((log10(T2))-(log10(T1)));
    A=(B*log10(T1))+(log10(log10(Z1)));

    Z=10^(10^(A-(B*(log10(T)))));
    VcSt=(Z-0.7)-exp(-0.7487-(3.295*(Z-0.7))+...
```

```
    (0.6119*((Z-0.7)^2))-(0.3193*((Z-0.7)^3)));
end

end
```

wearscar.m

```
function [a,kellip]=wearscar(wear,dx,Ra0)

th=Ra0/2;
Xfct=max(wear); var=find(Xfct>th);
if length(var)>1
    deltX=(max(var)-min(var)+1)*dx;
else
    deltX=dx;
end
Xfct=max(wear'); var=find(Xfct>th);
if length(var)>1
    deltY=(max(var)-min(var)+1)*dx;
else
    deltY=dx;
end
a=(deltX+deltY)/2; kellip=deltX/deltY;
if kellip>1
    kellip=1/kellip;
end

end
```

Introduction:

The purpose of this model is to numerically simulate with finite difference the process of sliding contact similar to what occurs during an ASTM D-4172 four-ball test. It was written in the Matlab programming language. By running this simulation, it is feasible to numerically predict the evolution of wear from sliding contact in a lubricated four-ball test.

Definition of Files:

- WearStudy.m:
    - This is the main program script for the model, running through all of the steps to determine the wear rate and total wear
    - This script can be converted to a function for a parametric study, if needed
    - Function calls on the "gethfct.m" function to determine the film thickness profile
- gethfct.m:
    - This function is used to iterate for the film-thickness profile
    - Function calls on the "findP.m" function to determine the pressure as it iterates for the film thickness profile
    - The function takes the guess for the pressure, predicts the film thickness, determines the pressure for the given film-thickness profile, and use the pressure to adjust the film-thickness; this iteration runs until there is convergence on the lubricant film-thickness and pressure
    - Output:
        - hOut: output final lubricant film-thickness profile
        - PfctOut: output final lubricant film pressure profile
- findP.m:
    - Determines the pressure for an input lubricant film-thickness profile
        - Assumes the film thickness is constant within the function
    - Uses a "guess" pressure for the nearest-neighbor pressure values to solve for the new pressure value at a given finite difference node
        - Iteration is used until the function converges on a pressure profile
    - Function calls on the "Roelands.m" function to determine the pressure-viscosity exponential coefficient, to calculate the true pressure as a function of the "guess" pressure during the iterative step
    - Output:
        - PfctOut: the output pressure function

- Roelands.m:
  - Calculates the pressure-viscosity exponential increase
    - $\mu = \mu_0 e^{\alpha * P}$
  - Determines the change in viscosity as a function of pressure
  - Calls on the "GetBeta" function
  - Output:
    - AP: the $\alpha*P$ term as a function of pressure
- GetBeta.m:
  - Calculates the viscosity-temperature exponential decay coefficient
    - $\mu(T) = \mu_0 e^{\beta(T - T_0)}$
  - Uses experimental data for the lubricant oil embedded into the function
  - Output: beta
- ViscFct.m:
  - Determines the kinematic viscosity for a given input temperature (in Centigrade)
  - Uses experimental viscosity-temperature data embedded in the function
  - Output:
    - VcSt: the kinematic viscosity in centistokes
- wearscar.m:
  - Determines the wear profile, and calculates the wear scar size and ellipticity
  - Wear is recognized when the wear exceeds the RMS surface roughness
  - The ellipticity consistently remains 1 throughout the model
  - Output:
    - a: the radius of the wear scar
    - kellip: the ellipticity of the wear scar (consistently 1)

Layout of the Code:

- Input Parameters
  - This is where the material and simulation parameters are inputted by the user
  - The first set are separated, as sometimes Matlab functions can be built with this information
    - These parameters, versus the function line, can be commented out if the script will be used as a function
    - Tb=59; % Bulk Lubricant Temperature (Centigrade)
      - This is the bulk lubricant oil temperature
      - The model will calculate localized heating from the pressure
    - maxdepth=5e-8; % Maximum depth of wear per cycle
      - The time step will adjust itself to ensure no more than this much wear (in meters of depth) will occur in a given time step
    - maxdt=1.0e0; % Maximum time step per cycle
      - The maximum time step in seconds, if a longer time step is calculated for the maximum wear depth per time step
    - Mx=201; % X and Z Nodes
      - The number of finite difference nodes in the X and Y direction (for Mx^2) nodes total
    - W0 = 88; % The load (in lbs) of the 4-ball test
    - Ra0=15.0e-8; % Initial surface roughness of ball bearing
    - nd = 0; % weight fraction of diamond nanoparticles
      - Set to 1e-4 for the standard 0.01% weight concentration
- Other model parameters
  - totaltime=3600; % Total Wear Time (seconds)
    - The total time (in seconds) of the sliding contact of the four-ball test
  - Ey=210e9; % Young's Modulus of the ball-bearing material (Pa)
  - poisson=.3; % Poisson Ratio of ball-bearing material
  - R = 0.25; % Radius of ball bearings in inches
    - Code will convert it to metric later
  - RPM=1200; % Speed of spindle in rpm
  - COF=0.10; % COF of interest

  - dt0 = 0e0; % Initial starting time-step
  - Bw=0.0014; % Bearing width (meters)
    - This is the length of the domain being analyzed
    - If it is made larger, the finite difference units will have lower resolution unless more nodes are added (which increases computational resources exponentially)
  - UTC=5e8; % Ultimate Tensile Strength of Steel (Pa)
  - SC = 0.6; % Ratio of ultimate strength in shear over ultimate tensile stress
  - Ea=-41e-21; % Diamond Nanoparticle Activation Energy (J)
  - K0=900; % Therman Conductivity Constant
  - k_boltz=1.38e-23; % Boltzman Constant (Joules / Kelvin)
- Input of ball-bearing material properties
  - k=46.6; % Thermal Conductivity of Material (W/m-K) – Steel
  - rho=7810; % Material Density (kg/m^3) – Steel
  - Cp=475; % Specific Heat of Material (J/kg-K) – Steel
- Input of diamond nanoparticle material properties
  - k_d=2190; % Thermal Conductivity of Material (W/m-K) – Diamond
  - rho_d=3530; % Material Density (kg/m^3) – Diamond
  - Cp_d=519; % Specific Heat of Material (J/kg-K) – Diamond
- Input of oil thermal properties
  - k_oil=0.140; % thermal conductivity of oil (W/m-K)
  - Cp_oil=2000; % specific heat of oil (J/kg-K)
  - rho_oil=905.75; % Material Density (kg/m^3) – Oil

- Calculate Parameters from Input
  - Make *Mx* an odd number
    - This assures there is a finite difference node that is at the center of the domain
    - ```
      if mod(Mx,2)==0
          Mx=Mx+1;
      end
      ```
  - dx=Bw/Mx;
    - The distance increment (meters) between each finite difference node
      ```

- Xfct=linspace(-Bw/2,Bw/2,Mx);
  - Generates distance function, used to calculate the oil film thickness
- W=W0*4.44822162/3; % Divide by 3 and convert to Newtons
  - Convert the force in pounds to Newtons, and divides by 3 because the force is evenly distributed over 3 ball bearings
- R=R*2.54/100;
  - Converts the radius of each ball bearing from inches to meters
- Rp=R/2;
  - $\frac{1}{R'}=\frac{1}{R_A}+\frac{1}{R_B}$, and $R_A=R_B=R$, therefore $R'=\frac{R}{2}$
  - 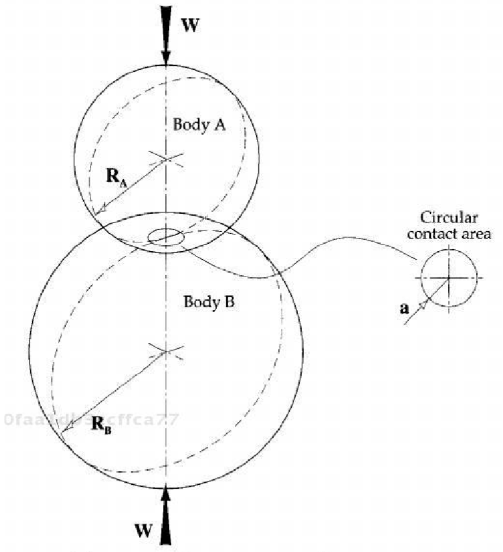

  - Reduced Radius, for Hertz Contact calculations
- Eyr=Ey/(1-(poisson^2));
  - $\frac{1}{E'}=\frac{1}{2}[\frac{1-v_A^2}{E_A}+\frac{1-v_B^2}{E_B}]$, and $E_A=E_B=E$, $v_A=v_B=v$, therefore $E'=\frac{E}{1-v^2}$
  - Calculates the reduced Young's modulus, for solving Hertzian contact equations
- aHertz=((3/2)*W*Rp/Eyr)^(1/3);
  - Uses Hertz's contact equations to calculate the radius of the area of contact, assuming all elastic deformation
  - $a=(\frac{3WR'}{2E'})^{\frac{1}{3}}$

- omg=RPM*(2*pi/60);
  - Converts the top-ball speed from revolutions per minute to radians per second
- U=0.5*omg*R; % Contact Speed (m/s)
  - Calculates the linear speed of sliding contact at the full specified speed
- ndx=nd*(1e4);
  - Determines the equivalent mass ratio of the 0.01% diamond nanofluid solution to neat mineral oil necessary to get the equivalent mass ratio of the lubricant being simulated
- k_nd=(1+(K0*exp(Ea/(k_boltz*(Tb+273.15)))))*k_oil;
  - Determines the thermal conductivity of the 0.01% weight concentration diamond nanoparticle solution only
  - Based on experimental studies, and follows an Arrhenius-like temperature dependence
- k_lub=(k_oil*(1-ndx))+(k_nd*ndx);
  - Thermal conductivity of lubricant with diamond nanoparticles
  - Derived as an average of mass functions of the neat mineral oil and the 0.01% weight concentration of diamond nanoparticles, to achieve the desired concentration of interest
- Cp_lub=(Cp_oil*(1-nd))+(Cp_d*nd);
  - Specific heat of lubricant with diamond nanoparticles (derived analytically)
- rho_lub=1/(((1-nd)/rho_oil)+(nd/rho_d));
  - Density of lubricant with diamond nanoparticles (derived analytically)
- TD=k/(rho*Cp); % Thermal Diffusivity (m^2/s)
  - The thermal diffusivity of the ball bearing, calculated from the input conductivity, heat capacity, and density
- TD_lub=k_lub/(rho_lub*Cp_lub); % Thermal Diffusivity of oil (m^2/s)
  - Uses lubricant parameters to allow for mixing of diamond nanoparticles
- kellip=1; % Elliptical Parameter = a/b
  - The initial ellipticity of the contact area
- oo = ceil(Mx/2);
  - Find the array position in the center of the contact area
  - Mx will consistently be an odd number

- Calculate the assumed added length W_P (meters) to take into consideration the shear yield stress of the ball bearing material (Greenwood Williamson theory)
  - $W_P = R'\left(\frac{G_{yield}}{E_y'}\right)^2$
    - where G_{yield} is the shear yield strength of the ball-bearing material (Pa)
- Maximum Hertzian pressure
  - $P_{Hertzian} = \frac{3}{2}\frac{W}{\pi a_{Hertz}^2}$
- Calculate the Hertzian deflection
  - $\delta_{Hertz} = \left(\frac{9}{16}\frac{W^2}{Ey'^2 R'}\right)^{\frac{1}{3}}$
- Calculate the ratio of deflection for pressure
  - $K_h = \frac{P_{Hertzian}}{\delta_{Hertz}}$
  - Uses the Winkler Mattress model to calculate the elastic deformation for a given fluid pressure by this ratio
  - 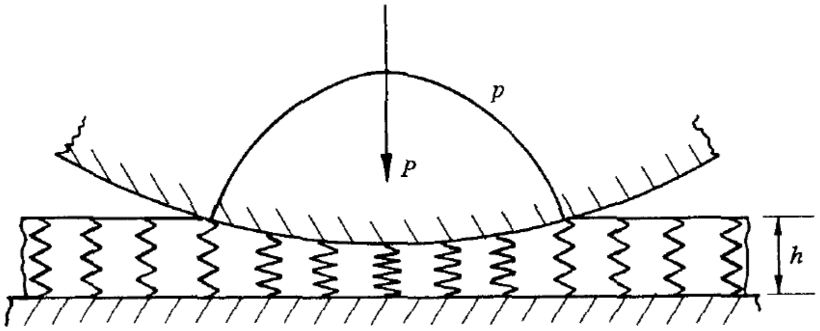

- Calculate the normalized radius R_w
  - To find the equivalent reduced radius, to normalize the dimensionless film thickness
    - To take into account that the contact is stationary sliding; Hamrock-Downson assumes moving rolling contact
  - Rw=Rp*((2*pi*R/(0.5*Bw/4.5))); $R_w = R'\frac{2\pi R}{0.5\frac{B_w}{4.5}}$
  - Ratio of the length of a revolution of the ball, over the approximate wear scar length
    - 4.5 is the minimum ratio of wear scar over total domain width (required to meet the Swift-Steiber boundary condition)

- Calculate the pressure-viscosity coefficient (See viscosity section for details)
  - $\alpha_{PVC} = 1.216 + 4.143(\log_{10} v)^{3.0627} + (\frac{2.848}{10^4} b_0^{5.1903})(\log_{10} v)^{1.5976} + 3.999 \rho_{density}^{0.1162} (\log_{10} v)^{3.0975}$
    - where v is in cSt, and b_0 is the ASTM slope coefficient times 5
  - For this oil, the ASTM slope coefficient b_0 is found to be 0.6363
  - The viscosity is calculated with the viscosity function (see Viscosity section)
  - This coefficient is used to find the viscosity changes with pressure following Barus' law:
    - $v = v_0 \exp[\alpha_{PVC} P]$
    - Breaks down when P < 0.5 Gpa
      - Roeland's theory is used for the Reynolds Equation Solver, where the pressure can exceed this level (See Roeland's Equation section)

- Calculate the initial minimum and central film thickness (see loop for details of equation)
  - The minimum film thickness is used in the iterative solver to find the film thickness function
    - Determined from the Hamrock Dowson empirical equations
    - $h_{min} = 3.63 R_w (\frac{\mu_0 U}{E_y' R'})^{0.68} (\alpha_{PVC} E_y')^{0.49} (\frac{W}{E_y' R'^2})^{-0.073} (1 - \exp[-0.68 \kappa_{ellipse}])$
  - Calculated at the beginning of each time-step throughout the simulation
    - Instabilities may occur if changes are too dramatic
      - With increasing temperature, viscosity decreases, and thus the minimum film thickness would decrease
      - With the minimum film thickness decreasing (such as from a temperature increase), the thermal resistance decreases (the oil is an insulator), and thus the temperature decreases
      - As a result of the temperature increase, the temperature decreases, which can cause numerical instabilities unless iteration for a proper average temperature is found
    - This new version assumes steady temperatures throughout each time-step
      - The only parameter that changes this minimum film thickness is the wear scar radius
      - As the wear scar diameter increases, the contact area increases, thus the friction heating density decreases, thus the temperature decreases, and thus the minimum film thickness increases, resulting in less wear
      - Temporally the wear rate decreases slowly in time (after initial running in)

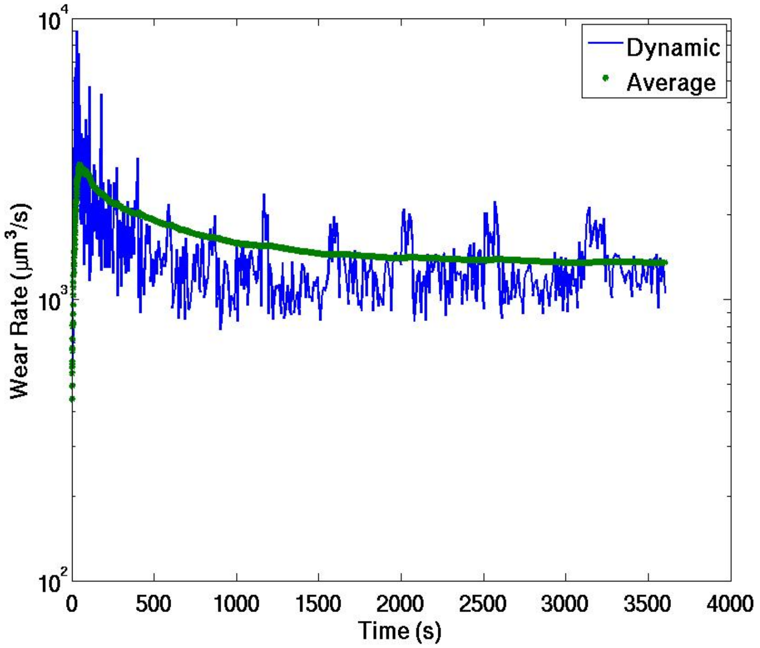

-

- First step is to calculate the flash temperature heating at the surface of the ball bearing
  - If Pecelt Number L < 0.1
    - $T_f = \frac{\mu_{COF} W U}{4 k a}$, where μ_{COF} is the coefficient of friction
    - Friction is considered a stationary heat source
  - If Pecelt Number 0.1 < L < 5
    - $T_f = [0.35 + (5 - L)\frac{0.5}{4.9}] \frac{\mu_{COF} W U}{4 k a}$
    - Friction heating is considered a slow-moving heat source
  - If Pecelt Number L > 5
    - $T_f = \frac{0.308 \mu_{COF} W U}{4 k a} \sqrt{\frac{\alpha}{U a}}$
    - Friction heating is considered a fast-moving heat source
- Next step is to calculate the temperature distribution within the oil film
  - For steady-state heat transfer with heat generation (effectively from the friction)
    - $\frac{d^2 T}{dx^2} = \frac{-\dot{g}}{k_{lub}} \rightarrow \dot{g} = \frac{\dot{Q}}{\pi a_{Hertz}^2} = \frac{\mu_{COF} W U}{\pi a_{Hertz}^2}$

- $T(x) = \frac{\dot{g}}{2k_{lub}}[hx - x^2] + T_f$
- $T_{avg} = 0.1665 \frac{\dot{g}}{2k_{lub}} h^2 + T_f$
  - where $\dot{g}$ is the equivalent heat generation (W/m^3), k_{lub} is the thermal conductivity of the oil, and h is the film thickness
- The next step is to use this average temperature to calculate the viscosity
  - See viscosity section
  - The bulk-temperature is used for the oil film thickness to start the iterations
- When a viscosity is determined, a central film thickness is estimated to calculate the new temperature profile
  - An analytical equation based on empirical data is used:
  - $h_c = 2.69 R_w \left(\frac{\mu_0 U}{E_y' R'}\right)^{0.67} (\alpha_{PVC} E_y')^{0.53} \left(\frac{W}{E_y' R'^2}\right)^{-0.067} (1 - 0.061 \bullet \exp[-0.73 \kappa_{ellipse}])$

- The iterations occur until there is convergence, looping through the following steps
  - Take the most recent central film thickness based on Dowson's equation
    - For the initial start of the iterative loop, use the viscosity estimated at the bulk temperature to estimate this thickness
  - Use the film thickness to determine the increase in oil film temperature
  - Determine the total film temperature by adding the flash temperature increase and the bulk temperature
  - Calculate the new viscosity based on the newly calculated lubricant temperature
  - Calculate the new central film thickness
  - If the error between the latest film thickness and the previous thickness is less than 1%, end the loop, and utilize this temperature and viscosity throughout the simulation
    - After 1000 iterations, the loop brakes and an alert is prompted

- Determine the minimum film thickness from the viscosity calculated by the converged temperature of the oil film, and use this initial minimum thickness throughout the simulation

- Calculate the viscosity
  - The viscosity is found through a separate viscosity Matlab function
  - Coefficients are first found before the time-steps start
    - It is necessary to know the viscosity of the lubricant at two temperature points, and for this to be declared; for example
      - T1=297.15; v1=638;
      - T2=363.15; v2=25;
    - In the separate function, the *A* and *B* terms are found for the two closest experimental data points, to reduce interpolation error when the theoretical viscosity is found
    - Find the value of *Z* at these temperatures
      - $Z = v + 0.7 + \exp(1.47 - 1.84\,v - 0.51\,v^2)$
      - ν is in cSt or mm^2/s
    - Find the coefficients of viscosity for the lubricating oil
      - $\log_{10}\log_{10} Z = A - B \log_{10} T$
      - $B = \frac{\log_{10}(\log_{10} Z_1) - \log_{10}(\log_{10} Z_2)}{\log_{10} T_2 - \log_{10} T_1}$, or $B = \frac{\log_{10}(\log_{10} Z_1) - \log_{10}(\log_{10} Z_2)}{\log_{10} T_2 - \log_{10} T_1}$
      - $A = \log_{10}\log_{10} Z_i + B \log_{10} T_i$, where *i* could be either 1 or 2
  - At each time step, find the new dynamic viscosity
    - Calculate the kinematic viscosity
      - Z = 10^(10^ $(A - B \log_{10} T_F)$)
      - $v = (Z - 0.7) - \exp[-0.7487 - 3.295(Z - 0.7) + 0.6119(Z - 0.7)^2 - 0.3193(Z - 0.7)^3]$
        - *v* is in cSt, T_f is in Kelvin
      - To calculate changes in viscosity
    - Convert to dynamic viscosity
      - $\mu(Pa - s) = \frac{v(cSt)}{10^6} \rho_{density}$
- Declare the arrays
  - In Matlab, if arrays are not pre-declared (as empty matrices), then creating and recreating the matrix size dynamically will dramatically increase the computational time

- ◦ Most matrices are set as Mx by Mx arrays of zero, where Mx is the number of finite difference points in the X and Z direction
  - wear=zeros(Mx,Mx);
  - indent=zeros(Mx,Mx);
- ◦ Indent is an array to represent the changing depth of the oil thickness as a result of the curvature of the ball bearing
  - $r'=\sqrt{X^2+Y^2}=R\sin\theta$, and $R-I=R\sin\theta$, therefore
    - $I=R[1-\cos(\sin^{-1}\frac{r'}{R})]$
  - 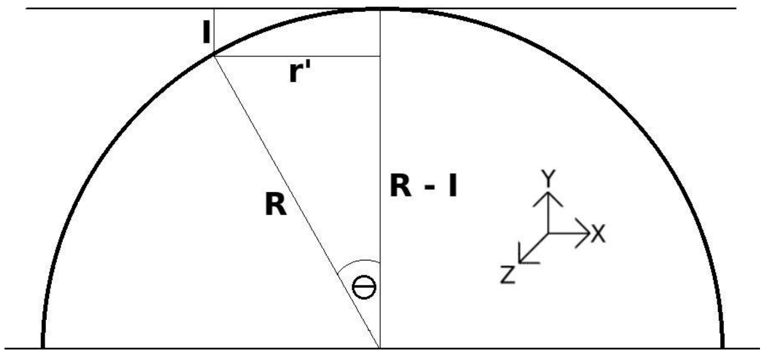

- ◦ Rfct
  - Determines the distance from the center of the domain to the location of each finite difference node
  - Rfct(ii,jj)=sqrt((Xfct(ii)^2)+(Yfct(jj)^2));
  - This is to be used for determining when a finite difference node is within the domain of the wear scar
- ◦ Determine the basis of the pressure function starting with Hertzian pressure
  - Determine the Hertzian pressure function
    - $P_{Hertzian}(r)=\frac{3}{2}\frac{W}{\pi a_{Hertz}^2}[1-(\frac{r}{a_{Hertz}})^2]$
  - Iterate the Reynolds solver to find the correct no-wear pressure distribution of the oil
    - See chapter on deflection and film thickness model

- Set up parameters for the start of the simulation
  - ◦ Set *a* as the radius of the contact area, starting at the Hertzian minimum
  - ◦ Set *TS* (the time step count) at 0

- Set *time = 0* at the start of full spindle acceleration
- Set *dt* to *dt0*

- Run the simulation (for each time step)
  - Calculate the new wear scar radius
  - Calculate the lubricant film temperature, lubricant viscosity, and the minimum elastohydrodynamic film thickness
  - Solve the Reynold's Equation with finite difference to determine the detailed lubricant profile
  - Calculate the asperities-film thickness ratio profile and resulting wear rate
  - Adjust the time-step
  - Calculate the total wear
- Save data

<u>At each time-step</u>:

- Calculate the size of the wear scar
  - Adjust scar diameter based on where there is some wear
  - If the wear scar is smaller than the Hertzian radius, adjust to the Hertzian radius
    - Hertzian is considered a valid assumption for a low or no-wear contact with an oil film thickness (ex. Grubin)
- Calculate the minimum film thickness
  - Uses Hamrock Dowson film-thickness
  - This will adjust as the temperature increases, and thus the atmospheric pressure (μ_0) decreases
- Calculate the oil thickness function
  - Iterate for the proper film thickness:
    - Determine the deflection based on oil pressure, utilizing the Winkler Mattress model
      - $\delta(x,z) = \frac{P(x,z)}{K_h}$
    - Determine the oil film thickness
      - $h(x,z) = 2 \bullet I(x,z) + \delta(x,z) + Wear(x,z) - min[2 \bullet I(x,z) + \delta(x,z) + Wear(x,z)] + h_{min}$
      - This assures that the minimum film thickness is the calculated minimum film thickness
      - In this equation and model *Wear(x,z)* is in meters, not volume

  - Determine the pressure with the Reynolds solver
  - Adjust the deflection function δ, and repeat the iteration until there is convergence
    - Require a minimum of 5 iterations, and a maximum of 25 iterations
- Reynolds Solver
  - If the film thickness is very thin (at the area of contact), it is reasonable to assume there is negligible Y directional changes
  - The Reynolds Equation:
    - $$\frac{\partial}{\partial x}\left(\frac{\rho h^3}{\mu}\frac{\partial P}{\partial x}\right)+\frac{\partial}{\partial z}\left(\frac{\rho h^3}{\mu}\frac{\partial P}{\partial z}\right)=\frac{\partial}{\partial x}[6\rho h(U_x)]+\frac{\partial}{\partial z}[6\rho h(U_z)]+12\frac{d}{dt}(\rho h)$$
  - If the flow is 1D, the Reynolds equation can be treated as 1D, where
    - $$\frac{\partial P}{\partial z}=\frac{\partial}{\partial z}\left(\frac{\rho h^3}{\mu}\frac{\partial P}{\partial z}\right)=0$$
  - and therefore:
    - $$\frac{\partial}{\partial x}\left(h^3\frac{\partial P}{\partial x}\right)=6\mu U\frac{\partial h}{\partial x}$$
      - Unidirectional Reynold's Equation
      - This equation can be used for both Pressure and the Grubin reduced Pressures
  - This can be derived into:
    - $$\frac{\partial P}{\partial x}=6\mu U\frac{h-h_c}{h^3}$$
    - where h_c is the upper film thickness where $\frac{\partial P}{\partial x}=0$, typically at the center of the region of contact
  - Convert the pressure differential into a discrete series with Taylor Series Expansion
    - $$P(X_{i+1})=P(X_i)+P'(X_{i+1})(X_{i+1}-X_i)+\bar{O}(\Delta X^2)$$
    - $$P'(X_i)=\frac{P(X_{i+1})-P(X_{i-1})}{2\Delta x}$$
    - $$P''(X_i)=\frac{P(X_{i+1})-2P(X_i)+P(X_{i-1})}{\Delta x^2}$$

- Using this, we can write the Reynold's equation in discrete linear form
  - $h^3 \frac{\partial^2 P}{\partial x^2} + 3h^2 \frac{\partial P}{\partial x}\frac{\partial h}{\partial x} = 6\mu U \frac{\partial h}{\partial x}$
  - $h^3 \frac{P(X_{i+1}) - 2P(X_i) + P(X_{i-1})}{\Delta x^2} + 3h^2 \frac{\partial h}{\partial x} + \frac{P(X_{i+1}) - P(X_{i-1})}{2\Delta x} = 6\mu U \frac{\partial h}{\partial x}$
  - $P_{i-1}\left(\frac{h^3}{\Delta x^2} - \frac{3h^2}{2\Delta x}\frac{\partial h}{\partial x}\right) + P_i\left(\frac{-2h^3}{\Delta x^2}\right) + P_{i+1}\left(\frac{h^3}{\Delta x^2} + \frac{3h^2}{2\Delta x}\frac{\partial h}{\partial x}\right) = 6\mu U \frac{\partial h}{\partial x}$
  - $(P_{i-1} W_i) + (P_i a_i) + (P_{i+1} E_i) = B_i$
- This same 1D equation can be converted to 2D for this simulation
  - $(P_{i-1,j} W_{i,j}) + (P_{i+1,j} E_{i,j}) + (P_{i,j-1} S_{i,j}) + (P_{i,j+1} N_{i,j}) + (P_{i,j} a_{i,j}) = B_{i,j}$
  - $W_{i,j} = \left(\frac{h^3}{\Delta x^2} - \frac{3h^2}{2\Delta x}\frac{\partial h}{\partial x}\right) = \frac{h(i,j)^3}{\Delta x^2} - \frac{3h(i,j)^2}{2\Delta x}\left(\frac{h(i+1,j) - h(i-1,j)}{2\Delta x}\right)$
  - $E_{i,j} = \left(\frac{h^3}{\Delta x^2} + \frac{3h^2}{2\Delta x}\frac{\partial h}{\partial x}\right) = \frac{h(i,j)^3}{\Delta x^2} + \frac{3h(i,j)^2}{2\Delta x}\left(\frac{h(i+1,j) - h(i-1,j)}{2\Delta x}\right)$
  - $S_{i,j} = \left(\frac{h^3}{\Delta z^2} - \frac{3h^2}{2\Delta z}\frac{\partial h}{\partial z}\right) = \frac{h(i,j)^3}{\Delta z^2} - \frac{3h(i,j)^2}{2\Delta z}\left(\frac{h(i,j+1) - h(i,j-1)}{2\Delta z}\right)$
  - $N_{i,j} = \left(\frac{h^3}{\Delta z^2} + \frac{3h^2}{2\Delta z}\frac{\partial h}{\partial z}\right) = \frac{h(i,j)^3}{\Delta z^2} + \frac{3h(i,j)^2}{2\Delta z}\left(\frac{h(i,j+1) - h(i,j-1)}{2\Delta z}\right)$
  - $a_{i,j} = -2\frac{h(i,j)^3}{dx^2} - 2\frac{h(i,j)^3}{dz^2}$
- It is of course necessary to watch for boundaries, and leave out empty data for boundary nodes that do not have a node to a directional border
- The right-hand side of this equation can discretely be solved as:
  - $B_i = 6\mu U \frac{\partial h}{\partial x} = (6\mu U)\frac{1}{2}\left[\left(\frac{h(i,j+1) - h(i,j-1)}{2\Delta x}\right) + \left(\frac{h(i,j+1) - h(i,j-1)}{2\Delta z}\right)\right]$
- Iterate to solve for the normalized pressure based on the older function for P
  - $P_{i,j} = \frac{B_{i,j} - [(P_{i-1,j} W_{i,j}) + (P_{i+1,j} E_{i,j}) + (P_{i,j-1} S_{i,j}) + (P_{i,j+1} N_{i,j})]}{a_{i,j}}$
  - All finite difference nodes at the boundary are set to 0
    - Assumed that far from the wear scar the oil pressure is practically nonexistent
  - Require a minimum of 2 iterations, and a maximum of 1000

Dry Contact Pressure (Hertzian) **Oil Pressure (Reynolds)**

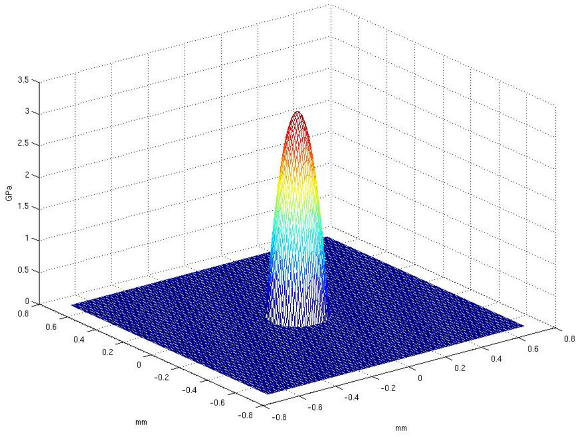

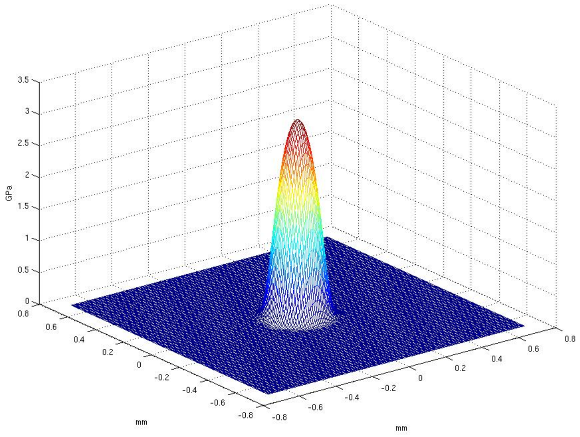

- Determine the pressure-viscosity effects
  - Barus' Law breaks down after 500 MPa; many pressures of interest exceed this level
  - Roelands equation is used to estimate the equivalent viscosity at each point when solving the Reynolds equation
    - $v = v_0 \exp[\alpha^x P]$
    - $\alpha^x P = \left(\frac{T_i - 138}{T_b - 138}\right)^{-S_0} \left[\left(1 + \frac{P}{1.9608 * 10^8}\right)^{Z_0} - 1\right][\log(\mu) + 9.67]$
    - $Z_0 = \frac{\alpha_{PVC}}{(5.1 \bullet 10^{-9})[\log(\mu_0) + 9.67]}$
      - Note: uses dynamic viscosity at the bulk-temperature (as the PVC coefficient was found at the bulk temperature)
      - All other equations use the viscosity at the lubricant film temperature
    - $S_0 = \beta \frac{T_b - 138}{[\log(\mu) + 9.67]}$
      - where β is obtained from the experimental viscosity-temperature data $v(T) = v(T_0)\exp[-\beta(T - T_0)]$
- Have a final determination for the pressure and film thickness function
  - The pressure can be used to determine the elastic deformation to calculate the film thickness
  - The film thickness will appear very flat at the area of contact

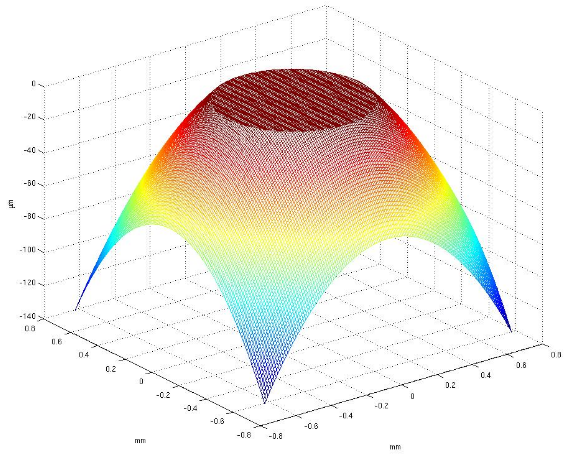

- Determine which data nodes are in the region of the wear scar
  - Done by taking the radius of each data node (found earlier), and determining which are less than the calculated wear scar radius
  - The wear scar radius is defined as the region where wear exceeds the original RMS surface roughness of the ball bearings
- Calculate the new RMS surface roughness *σ*
  - Calculated as the RMS of the different wear values as compared to the average wear depth for finite difference nodes within the wear scar
  - This total summation of the difference in wear is squared, summed up, divided by the total number of finite difference nodes in the wear scar, and square rooted
  - The original RMS value is then added to this newly calculated value, and it is multiplied by two, as there are two surfaces in contact (the sample ball bearing and the spinning ball bearing)
    - $$\frac{\sigma}{2}=\sigma_0+\sqrt{\frac{\Sigma\left[W(i,j)-\bar{W}\right]^2}{N_{nodes}}}$$
  - If σ is ever calculated to be less than σ_0, set $\sigma = \sigma_0$
  - This is not used for the numerical model, only for tracking and comparison to the optical profilometry data
- Find the ratio of asperity contact
  - A study was conducted to determine the ratio of wear for a given lambda-value (λ_W)
    - $\lambda_W=\frac{h+W_P}{\sigma}$, where σ is the standard deviation of the asperities height, h is the height of the oil film thickness, and W_P is the Greenwood Williamson height

- Monte Carlo was used to simulate the asperities
  - Asperities were represented by a series of random number from -1 to 1, which was then normalized by the standard deviation of the random number generator
    - This normalization was set so that a random height of 1 represents the RMS asperities height (σ)
      - No actual real values were used in this prior numerical Monte-Carlo study
    - The random series were multiplied by an odd exponential power when it was necessary to decrease the standard deviation
      - An odd power was necessary to ensure both negative and positive asperities
  - All asperities in excess of the specified lambda-value were removed

Before Wear

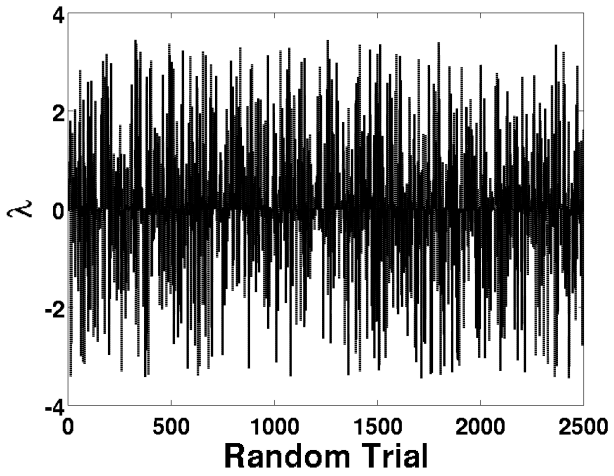

After Wear (λ_W = 1)

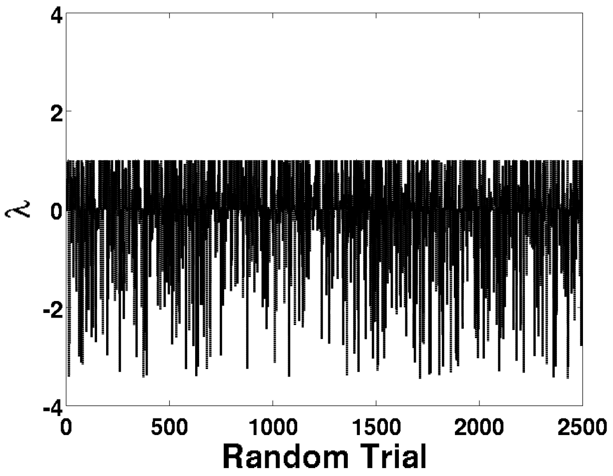

- Every time this happened, a count was recorded, to compare to the total number of random trials, to validate the area ratio
- Every time an asperities exceeded the λ_W value, the height in excess was recorded
    - This total height represents the total wear
    - Wear Volume: $V=\left(\frac{\Delta x^2}{N}\right)\sigma \Sigma_i^N \left(h_i - \lambda_W\right)$ (m^3)
    - where N is the total number of random asperities, and h_i represents all random normalized "asperities" in excess of λ_W; all other random asperities are set to zero
    - The total normalized wear was determined to follow (with reasonable error) an exponential decay function, where:
        - $V_N = 0.2763 \bullet \exp[-1.6754\, \lambda_W]$, where $V = V_n \bullet \Delta x^2 \bullet \sigma$ (m^3)
    - The wear rate at each finite difference node is simply: $\dot{V} = V_n \bullet \Delta x \bullet U \bullet \sigma$ (m^3/s)
- Calculate the time-step to ensure:
    - It is small enough that there would not be a wear depth increase exceeding the user specified maximum wear per time-step, where $dt = dW_{max} / \left(\frac{dW}{dt}\right)_{max}$
    - The calculated time-step is not longer than a user-specified maximum time-step duration
        - Once the max-wear calculated time-step exceeds the maximum time-step, the time-step is rounded down once so that the net time function is a clean integer of maximum time-steps
        - This is not necessary for anything except clean organization of data
- Save all the data within arrays

Chapter 1

Monte Carlo MatLab Source Code

- *runsim.m*: run the parametric Monte Carlo study
- *crunch.m*: function to complete a Monte Carlo study for a given λ_W-value
- *analyze.m*: determine the empirical equation (Eqn. **??**) for the normalized wear from the Monte Carlo empirical data
- *analyzeCOF.m*: determine the empirical equation (Eqn. **??**) for the coefficient of friction from the Monte Carlo empirical data

1.1 runsim.m

```
clear
tic

LambdaFct=0:0.01:3.0; ct=length(LambdaFct);
SubLoop=5;

WearRat=zeros(1,ct); RatErfc=zeros(1,ct);

for ii=1:(ct-1)
    Lambda=LambdaFct(ii);
    wearX=zeros(1,SubLoop); RatErfcX=zeros(1,SubLoop);
    for jj=1:SubLoop
        [wearX(jj),RatErfcX(jj)]=crunch(Lambda);
    end
    WearRat(ii)=mean(wearX); RatErfc(ii)=mean(RatErfcX);
end

save MCdata
toc
```

1.2 crunch.m

```
function [wear,RatErfc]=crunch(Lambda)

% Lambda = (hc + Wp) / Ra

Mx=1e4; pw=5;
if mod(pw,2)==0
    pw=pw+1;
end
StdRng=((2*rand(Mx,1))-1).^pw; StdRng=std(StdRng);
if Lambda>(1/StdRng)
    fprintf(['ALERT - Increase PW!!!!' '\n']);
end

Dat=((((2*rand(Mx^2,1))-1).^pw))/StdRng;
aa=find(Dat>Lambda); ct=length(aa);
RatErfc=ct/(Mx^2);

wear=0;
for ii=1:ct
    wear=wear+(Dat(aa(ii))-Lambda)*((1/Mx)^2);
end

end
```

1.3 analyze.m

```
clear

load MCdata

Decline=WearRat(ct-1)/WearRat(1);

coeff=-(1/LambdaFct(ct-1))*log(Decline);
Fct=WearRat(1)*exp(-coeff*LambdaFct);

plot(LambdaFct,WearRat,LambdaFct,Fct)

Error=(abs((Fct.*(WearRat.^-1))-1)); Error=mean(Error(1:(ct-1)));
```

1.4 analyzeCOF.m

```
clear

load MCdata

Decline=RatErfc(ct-1)/RatErfc(1);

coeff=-(1/LambdaFct(ct-1))*log(Decline);
Fct=RatErfc(1)*exp(-coeff*LambdaFct);

plot(LambdaFct,RatErfc,LambdaFct,Fct)

Error=(abs((Fct.*(RatErfc.^-1))-1)); Error=mean(Error(1:(ct-1)));
```

Temp	Trial #	Test Date	Test Date Trial #	Ball 1-Horizontal	Ball 1-Vertical	Ball 2-Horizontal	Ball 2-Vertical	Ball 3-Horizontal	Ball 3-Vertical	Average	Wear – Ball 1
29	1	06/18/14	6	0.66	0.66	0.66	0.66	.61	0.63	0.65	-2.62E+06
44	1	06/18/14	5	.659	0.65	.636	0.65	.66	0.7	0.67	-2.59E+06
51	1	06/24/14	2	.66	0.70	.67	0.69	0.74	0.71	0.71	-1.96E+06
59	1	06/18/14	4	.72	0.71	.74	0.74	0.73	0.71	0.72	-3.59E+06
67	1	06/24/14	1	.69	0.65	0.72	0.73	.66	0.67	0.69	-2.29E+06
74	1	03/01/13	1	0.618	0.605	0.619	0.624	0.608	0.623	0.62	-1.75E+06
29	2	06/21/14	2	0.67	0.71	.68	0.7	.64	0.64	0.68	-2.80E+06
44	2	06/20/14	1	0.644	0.64	.69	0.71	.68	0.69	0.67	-2.24E+06
51	2	06/24/14	4	.75	0.76	.73	0.74	.69	0.69	0.73	-4.58E+06
59	2	06/21/14	1	.72	0.74	.77	0.77	.80	0.82	0.78	-2.50E+06
67	2	06/24/14	3	.63	0.64	.60	0.61	.62	0.64	0.63	-2.03E+06
74	2	03/12/13	1	0.668	0.659	0.66	0.66	0.6	0.604	0.64	-2.40E+06

Temp (C)	Wear	Wear Rate	Diameter	RMS	RA	Max Wear Variation	Mean Wear Variation	Max Scar Variation	Mean Scar Variation	Max RMS Variation	Mean RMS Variation
29	-2.45E+006	679.9	0.667	7.607	6.287	0.224	0.145	0.000	0.000	0.181	0.097
44	-2.64E+006	734.0	0.669	8.109	6.763	0.179	0.117	0.000	0.000	0.096	0.059
51	-3.36E+006	933.8	0.720	9.264	7.676	0.418	0.224	0.000	0.000	0.274	0.129
59	-4.14E+006	1149.9	0.750	10.465	8.704	0.745	0.292	0.000	0.000	0.364	0.158
67	-2.35E+006	653.7	0.661	7.535	6.015	0.689	0.230	0.000	0.000	0.330	0.110
74	-1.96E+006	545.6	0.629	7.127	5.933	0.251	0.157	0.000	0.000	0.155	0.084

Wear – Ball 2	Wear – Ball 3	Variation – Ball 1	Variation – Ball 2	Variation – Ball 3	Mean Wear	Max Error	Surface RMS (um) 1	Surface Ra (um) 1	Surface RMS (um) 2	Surface Ra (um) 2	Surface RMS (um) 3
-2.53E+06	-1.90E+06	0.006	0.006	0.034	-2.35E+06	0.034	8.226	6.874	8.005	6.623	6.768
-2.17E+06	-2.96E+06	0.01116667	0.02416667	0.01433333	-2.57E+06	0.024	8.457	7.317	7.332	6.137	8.415
-3.03E+06	-3.84E+06	0.03	0.03	0.015	-2.94E+06	0.030	6.723	5.482	8.880	7.439	10.012
-4.08E+06	-2.77E+06	0.0075	0.0175	0.0025	-3.48E+06	0.018	9.858	8.291	10.631	8.984	8.559
-3.97E+06	-2.25E+06	0.0225	0.0325	0.0275	-2.84E+06	0.033	7.459	5.867	10.019	7.962	7.341
-1.90E+06	-1.69E+06	0.00466667	0.00533333	0.00066667	-1.78E+06	0.005	6.669	5.628	7.009	5.92	6.49
-2.89E+06	-1.93E+06	0.01	0.01	0.04	-2.54E+06	0.040	8.224	6.979	8.191	6.806	6.230
-2.90E+06	-2.99E+06	0.028	0.028	0.013	-2.71E+06	0.028	7.454	6.277	8.449	6.956	8.549
-3.92E+06	-2.85E+06	0.025	0.005	0.04	-3.78E+06	0.040	11.220	9.295	10.131	8.384	8.615
-4.68E+06	-7.22E+06	0.04666667	0.00666667	0.03333333	-4.80E+06	0.047	8.025	6.614	11.442	9.415	14.273
-1.54E+06	-2.03E+06	0.005	0.025	0	-1.87E+06	0.025	7.076	5.688	6.096	4.897	7.221
-2.46E+06	-1.60E+06	0.02166667	0.01816667	0.03983333	-2.15E+06	0.040	7.82	6.534	8.233	6.712	6.539

Wear Dev Ball 1	Wear Dev Ball 2	Wear Dev Ball 3	Wear Dev Ball 4	Wear Dev Ball 5	Wear Dev Ball 6	Scar Ball 1	Scar Ball 2	Scar Ball 3	Scar Ball 4	Scar Ball 5	Scar Ball 6
175500	86500	548500	354500	446500	514500	0.007	0.007	0.007	0.003	0.043	0.013
56333	473333	320667	400333	260667	348667	0.00983333	0.01883333	0.03283333	0.02483333	0.02483333	0.02116667
1405667	336667	479333	1217333	561333	515667	0.06	0.02	0.05	0.03	0.04	0.01
552500	63500	1366500	1642500	542500	3082500	0.02958333	0.03958333	0.00958333	0.02958333	0.00958333	0.02041667
59333	1620667	107333	322333	809333	322333	0.02875	0.01125	0.05875	0.03125	0.02125	0.06125
219333	64333	274333	433667	493667	369333	0.011	0.024	0.01	0.039	0.03	0.031

Surface Ra (um) 3	Avg Surface RMS (um)	Avg Surface Ra (um)	Cap Height h 1	Cap Height h 2	Cap Height h 3	Wear Assuming Flat 1	Wear Assuming Flat 2	Wear Assuming Flat 3	Flat Error 1	Flat Error 2	Flat Error 3
5.47	7.67	6.32	3.439E-005	3.439E-005	3.034E-005	-23553883	-23553883	-18334464	8.97974939	9.29513917	9.65479926
6.793	8.07	6.75	0.00003382	3.249E-005	3.651E-005	-22777140	-21017751	-26547247	8.80786541	9.69006493	8.95958383
8.267	8.54	7.06	3.651E-005	3.651E-005	4.152E-005	-26547247	-26547247	-34321407	13.5722121	8.77594938	8.93553933
7.171	9.68	8.15	4.038E-005	4.327E-005	4.095E-005	-32462726	-37258011	-33382344	9.05010487	9.14082701	12.0383498
5.753	8.27	6.53	3.545E-005	4.152E-005	3.492E-005	-25016938	-34321407	-24277130	10.9053782	8.63648882	10.8090515
5.45	6.72	5.67	2.951E-005	3.049E-005	2.990E-005	-17348001	-18512826	-17807115	9.94154783	9.74359274	10.5367547
4.969	7.55	6.25	3.760E-005	3.760E-005	3.233E-005	-28146853	-28146853	-20821525	10.0452723	9.72593398	10.7716115
7.095	8.15	6.78	3.274E-005	3.870E-005	3.705E-005	-21347877	-29817829	-27338259	9.52180076	10.2713845	9.14017351
7.188	9.99	8.29	4.504E-005	4.268E-005	3.760E-005	-40379599	-36258963	-28146853	8.81843164	9.24266199	9.88996941
11.75	11.25	9.26	4.210E-005	4.686E-005	5.187E-005	-35280188	-43693835	-53533675	14.1290299	9.33230138	7.41258308
5.921	6.80	5.50	3.183E-005	2.889E-005	3.133E-005	-20177366	-16620975	-19548295	9.93469515	10.7648801	9.62496046
5.356	7.53	6.20	3.476E-005	3.439E-005	2.860E-005	-24058428	-23553883	-16293255	10.0327056	9.58253973	10.2152072

RMS Ball 1	RMS Ball 2	RMS Ball 3	RMS Ball 4	RMS Ball 5	RMS Ball 6
0.61866667	0.39766667	0.83933333	0.61666667	0.58366667	1.37733333
0.34766667	0.77733333	0.30566667	0.65533333	0.33966667	0.43966667
2.5405	0.3835	0.7485	1.9565	0.8675	0.6485
0.60666667	0.16633333	1.90566667	2.43966667	0.97733333	3.80833333
0.07633333	2.48366667	0.19433333	0.45933333	1.43933333	0.31433333
0.45766667	0.11766667	0.63666667	0.69333333	1.10633333	0.58766667

Oil	Temp	Trial #	Ball 1-Horizontal	Ball 1-Vertical	Ball 2-Horizontal	Ball 2-Vertical	Ball 3-Horizontal	Ball 3-Vertical	Average
MO	29	1	0.66	0.66	0.66	0.66	.61	0.63	0.65
MO	44	1	.659	0.65	.636	0.65	.66	0.7	0.67
MO	51	1	.66	0.70	.67	0.69	0.74	0.71	0.71
MO	59	1	.72	0.71	.74	0.74	0.73	0.71	0.72
MO	67	1	.69	0.65	0.72	0.73	.66	0.67	0.69
MO	29	2	0.67	0.71	.68	0.7	.64	0.64	0.68
MO	44	2	0.644	0.64	.69	0.71	.68	0.69	0.67
MO	51	2	.75	0.76	.73	0.74	.69	0.69	0.73
MO	59	2	.72	0.74	.77	0.77	.80	0.82	0.78
MO	67	2	.63	0.64	.60	0.61	.62	0.64	0.63

Test Date	Test Date Trial #	Wear – Ball 1	Wear – Ball 2	Wear – Ball 3	Variation – Ball 1	Variation – Ball 2	Variation – Ball 3	Mean Wear	Max Error
06/18/14	6	-2.62E+06	-2.53E+06	-1.90E+06	0.006	0.006	0.034	-2.35E+06	0.034
06/18/14	5	-2.59E+06	-2.17E+06	-2.96E+06	0.01116667	0.02416667	0.01433333	-2.57E+06	0.024
06/24/14	2	-1.96E+06	-3.03E+06	-3.84E+06	0.03	0.03	0.015	-2.94E+06	0.030
06/18/14	4	-3.59E+06	-4.08E+06	-2.77E+06	0.0075	0.0175	0.0025	-3.48E+06	0.018
06/24/14	1	-2.29E+06	-3.97E+06	-2.25E+06	0.0225	0.0325	0.0275	-2.84E+06	0.033
06/21/14	2	-2.80E+06	-2.89E+06	-1.93E+06	0.01	0.01	0.04	-2.54E+06	0.040
06/20/14	1	-2.24E+06	-2.90E+06	-2.99E+06	0.028	0.028	0.013	-2.71E+06	0.028
06/24/14	4	-4.58E+06	-3.92E+06	-2.85E+06	0.025	0.005	0.04	-3.78E+06	0.040
06/21/14	1	-2.50E+06	-4.68E+06	-7.22E+06	0.04666667	0.00666667	0.03333333	-4.80E+06	0.047
06/24/14	3	-2.03E+06	-1.54E+06	-2.03E+06	0.005	0.025	0	-1.87E+06	0.025

52

Surface RMS (um) 1	Surface Ra (um) 1	Surface RMS (um) 2	Surface Ra (um) 2	Surface RMS (um) 3	Surface Ra (um) 3	Avg Surface RMS (um)	Avg Surface Ra (um)
8.226	6.874	8.005	6.623	6.768	5.47	7.67	6.32
8.457	7.317	7.332	6.137	8.415	6.793	8.07	6.75
6.723	5.482	8.880	7.439	10.012	8.267	8.54	7.06
9.858	8.291	10.631	8.984	8.559	7.171	9.68	8.15
7.459	5.867	10.019	7.962	7.341	5.753	8.27	6.53
8.224	6.979	8.191	6.806	6.230	4.969	7.55	6.25
7.454	6.277	8.449	6.956	8.549	7.095	8.15	6.78
11.220	9.295	10.131	8.384	8.615	7.188	9.99	8.29
8.025	6.614	11.442	9.415	14.273	11.75	11.25	9.26
7.076	5.688	6.096	4.897	7.221	5.921	6.80	5.50

T=59C

Oil	Time (s)	Trial #	Ball 1- Horizontal	Ball 1- Vertical	Ball 2- Horizontal	Ball 2- Vertical	Ball 3- Horizontal	Ball 3- Vertical	Average
MO	10	1	.370	0.355	.358	0.341	.351	0.338	0.345
MO	60	1	.385	0.383	.383	0.370	.385	0.376	0.376
MO	120	1	.403	0.388	.424	0.424	.385	0.392	0.401
MO	300	1	.437	0.435	.435	0.430	.448	0.430	0.432
MO	1800	1	.579	0.585	.560	0.560	.602	0.616	0.587
MO	3600	1	0.720	0.710	.74	0.740	.73	0.710	0.720
MO	10	2	.374	0.354	.358	0.343	.363	0.349	0.349
MO	60	2	.388	0.388	.376	0.363	.372	0.370	0.374
MO	120	2	.385	0.365	.397	0.388	.381	0.367	0.373
MO	300	2	.424	0.424	.460	0.455	.444	0.437	0.439
MO	1800	2	.556	0.570	.587	0.593	.570	0.587	0.583
MO	3600	2	.72	0.740	0.770	0.770	0.800	0.820	0.780

T=59C

Test Date	Test Date Trial #	Wear – Ball 1	Wear – Ball 2	Wear – Ball 3	Variation – Ball 1	Variation – Ball 2	Variation – Ball 3	Mean Wear	Max Error
06/30/14	2	-2.25E+004	-2.98E+004	-1.12E+004	0.018	0.005	0.000	-2.12E+04	0.018
07/01/14	1	-1.34E+005	-8.88E+004	-6.53E+004	0.008	0.000	0.004	-9.60E+04	0.008
07/01/14	2	-7.01E+004	-2.28E+005	-8.82E+004	0.006	0.023	0.013	-1.29E+05	0.023
06/30/14	3	-2.90E+005	-2.57E+005	-2.95E+005	0.004	0.001	0.007	-2.81E+05	0.007
06/30/14	1	-1.51E+006	-1.18E+006	-1.99E+006	0.005	0.027	0.022	-1.56E+06	0.027
06/18/14	4	-3.59E+006	-4.08E+006	-2.77E+006	0.005	0.020	0.000	-3.48E+06	0.020
07/04/12	7	-4.59E+004	-5.66E+003	-3.39E+004	0.015	0.002	0.007	-2.85E+04	0.015
07/04/12	6	-1.34E+005	-2.88E+004	-6.67E+004	0.014	0.004	0.003	-7.66E+04	0.014
07/04/12	5	-6.71E+004	-1.30E+005	-9.10E+004	0.002	0.019	0.001	-9.60E+04	0.019
07/04/12	4	-2.39E+005	-3.84E+005	-2.62E+005	0.015	0.019	0.002	-2.95E+05	0.019
07/04/12	3	-1.25E+006	-1.56E+006	-1.40E+006	0.020	0.007	0.005	-1.40E+06	0.020
06/21/14	1	-2.50E+006	-4.68E+006	-7.22E+006	0.050	0.010	0.030	-4.80E+06	0.050

Surface RMS (um) 1	Surface Ra (um) 1	Surface RMS (um) 2	Surface Ra (um) 2	Surface RMS (um) 3	Surface Ra (um) 3	Avg Surface RMS (um)	Avg Surface Ra (um)
0.434	0.253	0.537	0.328	0.280	0.174	0.42	0.25
1.440	1.042	1.052	0.715	0.787	0.56	1.09	0.77
0.715	0.55	1.896	1.486	0.905	0.674	1.17	0.90
2.170	1.75	1.964	1.555	2.152	1.777	2.10	1.69
6.158	5.075	5.144	3.994	7.299	6.011	6.20	5.03
9.858	8.291	10.631	8.984	8.559	7.171	9.68	8.15
0.67	0.43	0.146	0.10	0.590	0.34	0.47	0.29
1.510	1.05	0.419	0.27	0.937	0.62	0.96	0.65
0.742	0.54	1.250	0.96	1.024	0.76	1.01	0.75
1.865	1.51	2.656	2.24	1.946	1.64	2.16	1.79
5.502	4.61	6.136	5.13	5.779	4.80	5.81	4.85
8.025	6.614	11.442	9.415	14.273	11.75	11.25	9.26

T=51C

Oil	Time (s)	Trial #	Ball 1-Horizontal	Ball 1-Vertical	Ball 2-Horizontal	Ball 2-Vertical	Ball 3-Horizontal	Ball 3-Vertical	Average
MO	10	1	.365	0.355	.353	0.331	.366	0.353	0.35
MO	60	1	0.382	0.366	.375	0.363	.392	0.385	0.37
MO	120	1	0.379	0.351	.416	0.402	.396	0.376	0.38
MO	300	1	.446	0.448	.444	0.426	.433	0.433	0.44
MO	1800	1	.628	0.600	.582	0.598	.616	0.586	0.59
MO	3600	1	.66	0.700	.67	0.690	0.740	0.710	0.71
MO	10	2	.358	0.352	.361	0.352	.352	0.336	0.35
MO	60	2	0.385	0.370	.365	0.349	.390	0.381	0.37
MO	120	2	.394	0.374	.392	0.376	.406	0.394	0.38
MO	300	2	.430	0.424	.446	0.437	.444	0.444	0.44
MO	1800	2	.579	0.599	.602	0.613	.556	0.579	0.60
MO	3600	2	.75	0.760	.73	0.740	.69	0.690	0.73

Test Date	Test Date Trial #	Wear – Ball 1	Wear – Ball 2	Wear – Ball 3	Variation – Ball 1	Variation – Ball 2	Variation – Ball 3	Mean Wear	Max Error
06/26/14	3	-9.23E+03	-3.56E+03	-1.52E+04	0.014	0.004	0.013	-9.32E+03	0.014
06/26/14	2	-2.09E+04	-5.27E+04	-1.33E+05	0.000	0.005	0.015	-6.90E+04	0.015
06/26/14	1	-7.72E+04	-1.91E+05	-6.80E+04	0.012	0.032	0.009	-1.12E+05	0.032
06/25/14	3	-2.79E+05	-2.35E+05	-1.94E+05	0.011	0.001	0.003	-2.36E+05	0.011
06/25/14	1	-1.36E+06	-1.10E+06	-1.01E+06	0.019	0.005	0.006	-1.16E+06	0.019
06/24/14	2	-1.96E+06	-3.03E+06	-3.84E+06	0.030	0.030	0.015	-2.94E+06	0.030
06/26/14	6	-8.94E+03	-9.19E+03	-7.05E+03	0.008	0.010	0.003	-8.39E+03	0.010
06/26/14	5	-2.91E+04	-3.50E+04	-1.11E+05	0.006	0.014	0.014	-5.82E+04	0.014
06/26/14	4	-1.28E+05	-7.27E+04	-1.36E+05	0.003	0.003	0.019	-1.12E+05	0.019
06/25/14	4	-2.28E+05	-2.95E+05	-2.87E+05	0.008	0.007	0.009	-2.70E+05	0.009
06/25/14	2	-1.40E+06	-1.84E+06	-1.23E+06	0.008	0.011	0.030	-1.49E+06	0.030
06/24/14	4	-4.58E+06	-3.92E+06	-2.85E+06	0.025	0.005	0.040	-3.78E+06	0.040

Surface RMS (um) 1	Surface Ra (um) 1	Surface RMS (um) 2	Surface Ra (um) 2	Surface RMS (um) 3	Surface Ra (um) 3	Avg Surface RMS (um)	Avg Surface Ra (um)
0.211	0.136	0.186	0.133	0.239	0.177	0.21	0.15
0.536	0.345	0.761	0.519	1.479	1.086	0.93	0.65
0.901	0.684	1.765	1.34	0.658	0.489	1.11	0.84
2.064	1.628	1.837	1.481	1.601	1.23	1.83	1.45
5.361	4.246	4.714	3.703	4.433	3.44	4.84	3.80
6.723	5.482	8.880	7.439	10.012	8.267	8.54	7.06
0.207	0.143	0.227	0.17	0.183	0.125	0.21	0.15
0.398	0.297	0.491	0.339	1.299	0.981	0.73	0.54
1.326	1.023	0.763	0.587	1.295	1.002	1.13	0.87
1.837	1.523	2.145	1.725	2.051	1.675	2.01	1.64
5.684	4.752	6.776	5.664	5.097	4.171	5.85	4.86
11.220	9.295	10.131	8.384	8.615	7.188	9.99	8.29
